# Xpert MTB/RIF Ultra versus mycobacterial growth indicator tube liquid culture for detection of *Mycobacterium tuberculosis* in symptomatic adults: a diagnostic accuracy study

**DOI:** 10.1016/S2666-5247(24)00001-6

**Published:** 2024-04-09

**Authors:** Yingda L Xie, Christie Eichberg, Nchimunya Hapeela, Elizabeth Nakabugo, Irene Anyango, Kiranjot Arora, Jeffrey E Korte, Ronald Odero, Judi van Heerden, Widaad Zemanay, Samuel Kennedy, Pamela Nabeta, Mahmud Hanif, Camilla Rodrigues, Alena Skrahina, Wendy Stevens, Reynaldo Dietze, Xin Liu, Jerrold J Ellner, David Alland, Moses L Joloba, Samuel G Schumacher, Kimberly D McCarthy, Lydia Nakiyingi, Susan E Dorman

**Affiliations:** Department of Medicine, Rutgers New Jersey Medical School, Newark, NJ, USA; Department of Medicine, Medical University of South Carolina, Charleston, SC, USA; Division of Medical Microbiology and Institute for Infectious Diseases and Molecular Medicine, University of Cape Town, Cape Town, South Africa; Infectious Diseases Institute, Makerere University, Kampala, Uganda; Kenya Medical Research Institute, Center for Global Health Research, Kisumu, Kenya; Department of Medicine, Rutgers New Jersey Medical School, Newark, NJ, USA; Department of Medicine, Medical University of South Carolina, Charleston, SC, USA; Kenya Medical Research Institute, Center for Global Health Research, Kisumu, Kenya; Division of Medical Microbiology and Institute for Infectious Diseases and Molecular Medicine, University of Cape Town, Cape Town, South Africa; Division of Medical Microbiology and Institute for Infectious Diseases and Molecular Medicine, University of Cape Town, Cape Town, South Africa; Department of Medicine, Medical University of South Carolina, Charleston, SC, USA; FIND, Geneva, Switzerland; State TB Training and Demonstration Centre, New Delhi, India; PD Hinduja Hospital and Medical Research Centre, Mumbai, India; National Reference Laboratory, Republican Scientific and Practical Centre for Pulmonology and Tuberculosis, Minsk, Belarus; Department of Molecular Medicine and Hematology, Faculty of Health Science, School of Pathology, and the National Priority Program of the National Health Laboratory Service, Johannesburg, South Africa; Universidade Federal do Espirito Santo, Vitoria, Brazil; Henan Provincial Chest Hospital, Zhengzhou, China; Department of Medicine, Rutgers New Jersey Medical School, Newark, NJ, USA; Department of Medicine, Rutgers New Jersey Medical School, Newark, NJ, USA; Mycobacteriology Laboratory, Department of Microbiology, School of Biomedical Sciences, Makerere University, Kampala, Uganda; FIND, Geneva, Switzerland; US Centers for Disease Control and Prevention, Kisumu, Kenya; Infectious Diseases Institute, Makerere University, Kampala, Uganda; Department of Medicine, Medical University of South Carolina, Charleston, SC, USA

## Abstract

**Background:**

Xpert MTB/RIF Ultra (Ultra) is an automated molecular test for the detection of *Mycobacterium tuberculosis* in sputum. We compared the sensitivity of Ultra to that of mycobacterial growth indicator tube (MGIT) liquid culture, considered the most sensitive assay in routine clinical use.

**Methods:**

In this prospective, multicentre, cross-sectional diagnostic accuracy study, we used a non-inferiority design to assess whether the sensitivity of a single Ultra test was non-inferior to that of a single liquid culture for detection of *M tuberculosis* in sputum. We enrolled adults (age ≥18 years) with pulmonary tuberculosis symptoms in 11 countries and each adult provided three sputum specimens with a minimum volume of 2 mL over 2 days. Ultra was done directly on sputum 1, and Ultra and MGIT liquid culture were done on resuspended pellet from sputum 2. Results of MGIT and solid media cultures done on sputum 3 were considered the reference standard. The pre-defined non-inferiority margin was 5·0%.

**Findings:**

Between Feb 18, 2016, and Dec 4, 2019, we enrolled 2906 participants. 2600 (89%) participants were analysed, including 639 (25%) of 2600 who were positive for tuberculosis by the reference standard. Of the 2357 included in the non-inferiority analysis, 877 (37%) were HIV-positive and 984 (42%) were female. Sensitivity of Ultra performed directly on sputum 1 was non-inferior to that of sputum 2 MGIT culture (MGIT 91·1% *vs* Ultra 91·9%; difference −0·8 percentage points; 95% CI −2·8 to 1·1). Sensitivity of Ultra performed on sputum 2 pellet was also non-inferior to that of sputum 2 MGIT (MGIT 91·1% *vs* Ultra 91·9%; difference −0·8 percentage points; −2·7 to 1·0).

**Interpretation:**

For the detection of *M tuberculosis* in sputum from adults with respiratory symptoms, there was no difference in sensitivity of a single Ultra test to that of a single MGIT culture. Highly sensitive, rapid molecular approaches for *M tuberculosis* detection, combined with advances in genotypic methods for drug resistance detection, have potential to replace culture.

**Funding:**

US National Institute of Allergy and Infectious Diseases.

## Introduction

The Xpert MTB/RIF tests (Cepheid, Sunnyvale, CA) are automated, integrated, cartridge-based molecular assays intended for tuberculosis case detection and identification of rifampicin resistance.^1-3^ The original Xpert MTB/RIF assay (ie, Xpert) was endorsed by WHO in 2010.^4^ Xpert MTB/RIF Ultra (ie, Ultra) was designed to address several limitations of the original test. Most notably, Ultra incorporates two different multicopy amplification targets to increase test sensitivity.^5^ Analytical laboratory studies demonstrated approximately one log improvement in the lower limit of detection for Ultra compared with Xpert.^5^ A multicentre clinical diagnostic accuracy study confirmed that Ultra was more sensitive than Xpert overall as well as in a subgroup with paucibacillary pulmonary disease and a subgroup with HIV, although Ultra had lower specificity than Xpert.^6^ Other large clinical diagnostic accuracy studies have confirmed the high sensitivity of Ultra for the diagnosis of pulmonary tuberculosis in adults and children and for the diagnosis of extrapulmonary tuberculosis.^7-9^ WHO endorsed Ultra in 2017 and it has been rolled out in national tuberculosis programmes.^10^

Mycobacterial culture using liquid media has been the clinical gold standard for the diagnosis of active tuberculosis for about three decades. Nevertheless, mycobacterial culture has important practical and technical limitations. Growth in culture requires days to weeks and substantial laboratory infrastructure for test performance and biosafety. Cultures are subject to overgrowth by contaminating microorganisms and might yield no clinically useful information for some specimens. Preparation of respiratory specimens for culture requires exacting processing steps that can decrease viable mycobacteria, thereby reducing sensitivity.^11-13^ There are also *Mycobacterium tuberculosis* bacterial phenotypes that do not grow readily in routine culture media.^14,15^

The advent of highly sensitive rapid molecular tests raises the possibility of transforming the clinical diagnostic process by eliminating culture. In this study, we sought to test the hypothesis that, for pulmonary tuberculosis case detection, the sensitivity of a single sputum Ultra test is non-inferior to that of a single mycobacterial liquid culture performed from sputum using the BACTEC Mycobacterial Growth Indicator Tube (MGIT) automated system (BD Microbiology Systems, Sparks, MD, USA).

## Methods

### Study design and procedures

In this diagnostic accuracy study, we combined data from a cohort of newly enrolled, newly tested participants with data from a previously published cohort.^6^ For both cohorts, specimens and microbiological testing were prospectively conducted according to written protocols (appendix p 5) and data were collected on study-specific forms. In the original cohort, the primary study objective was to determine Ultra diagnostic accuracy versus a microbiological reference standard comprising multiple sputum cultures. Participants in the original cohort were enrolled at primary health centres and hospitals in eight countries (Belarus, Brazil, China, Georgia, India, Kenya, South Africa, and Uganda). The current study extended enrolment at sites in Kenya, South Africa, and Uganda, using the same case detection group eligibility criteria and specimen testing procedures as reported previously.^6^ Per the protocol, extension of enrolment was done explicitly to assess for non-inferiority of Ultra sensitivity versus MGIT.

**Figure 1:**
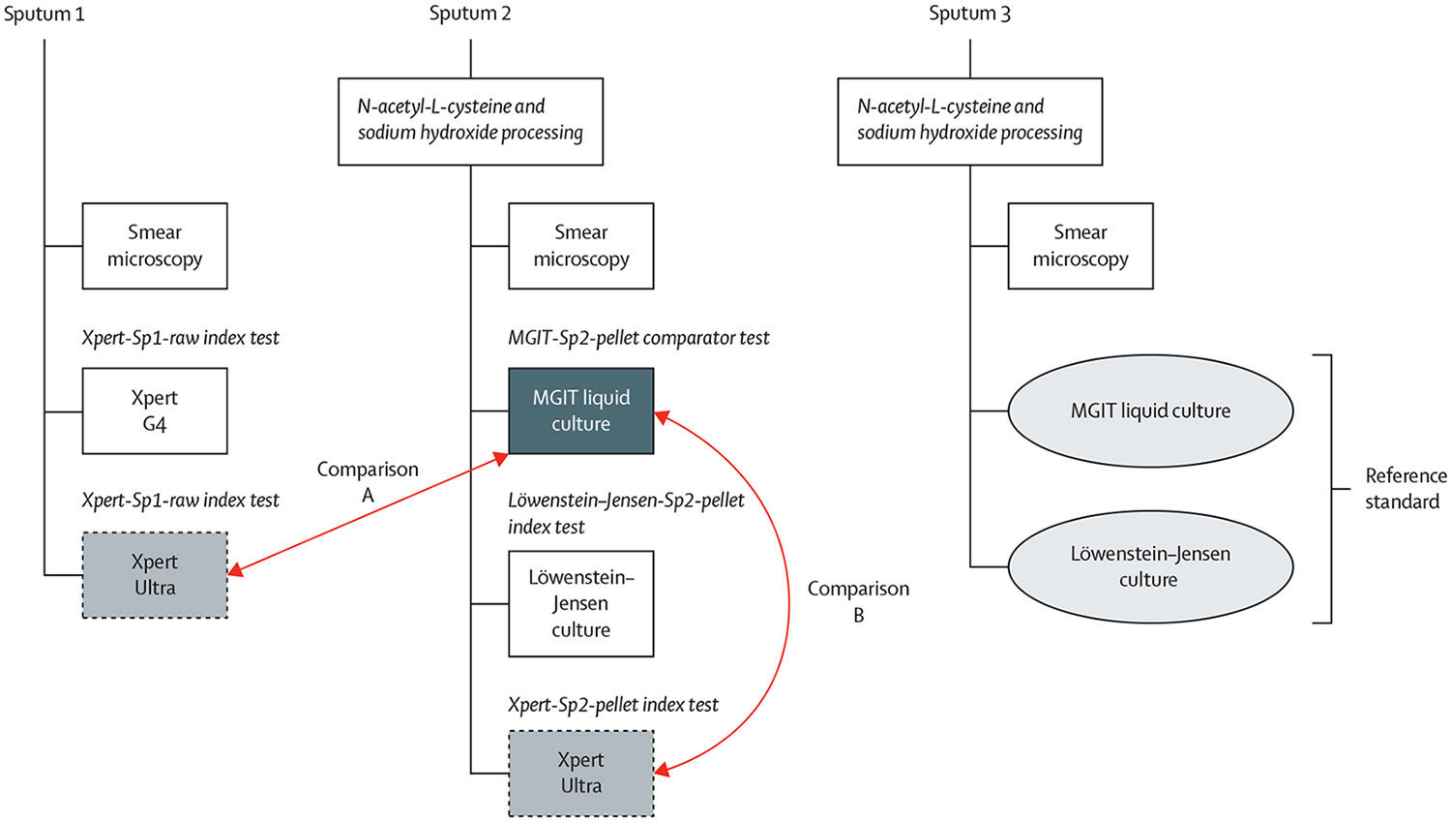
Schema for sputum testing and comparison of diagnostic accuracy of Ultra *vs* MGIT liquid culture The reference standard was the culture result from sputum 3; both MGIT and Löwenstein–Jensen culture results were used. The comparator test was the MGIT culture done on the resuspended pellet from sputum 2 (MGIT-Sp2-pellet). For primary comparison A, the index test was the Ultra test done directly on unprocessed sputum 1 (Ultra-Sp1-raw). For primary comparison B, the index test was the Ultra test done on the resuspended pellet from sputum 2 (Ultra-Sp2-pellet). MGIT=mycobacterial growth indicator tube liquid culture.

Study participants were adults (age ≥18 years) presenting with pulmonary tuberculosis symptoms of cough for at least 2 weeks plus at least one additional tuberculosis sign or symptom (eg, fever, night sweats, weight loss) and indication for pulmonary tuberculosis diagnostic evaluation per national tuberculosis programme guidelines. Participants formed a consecutive series (ie, all eligible participants were offered enrolment). Individuals were excluded as ineligible if they had received any tuberculosis antimicrobials within 6 months before enrolment or were enrolled into the multidrug resistance risk group of the previous cohort.^6^ Demographic information, medical history, and HIV status were recorded at enrolment. Classification of a participant as having had (versus not) previously treated tuberculosis was based on participant self-report. Participants were asked to provide three spontaneously expectorated sputum specimens with a minimum volume of 2 mL per specimen over 2 days. The interval between each sputum collection was at least 1 h, with the third sputum collection targeted as a morning sample on the second day. Tests were performed at on-site study laboratories according to written procedures (figure 1).

For sputum sample 1, smear microscopy was done using auramine-rhodamine staining and visualised bacilli burden was graded as negative, scanty, 1+, 2+, or 3+ by the laboratory microscopist.^16^ Xpert and Ultra assays were done by adding kit-provided sample reagent in a 2:1 dilution to the sputum sample and adding 2·0 mL of the resulting mixture to one Xpert cartridge and one Ultra cartridge. Samples were analysed using GeneXpert instruments with automated results readouts of *M tuberculosis* detected, *M tuberculosis* not detected, invalid, error, or no result. The semiquantitative scale for Ultra results was trace, very low, low, medium, or high. A result of detection (including trace) of *M tuberculosis* was considered positive regardless of semiquantitative results.

For sputum specimens 2 and 3, each specimen was digested with N-acetyl-L-cysteine and sodium hydroxide (final concentration 1·0–1·5%), which was concentrated using centrifugation, and the resulting pellet was resuspended in phosphate-buffered saline (pH 6·8) to a final volume of 2·0 mL.^17^ Smear microscopy was done using auramine–rhodamine staining (50 μL) of the pellet and graded as negative, scanty, 1+, 2+, or 3+. For cultures, 0·5 mL of the resuspended pellet was inoculated into a MGIT liquid culture vial that was incubated using the BACTEC 960 instrument (BD Microbiology Systems) and 0·2 mL of this was inoculated onto Löwenstein–Jensen medium (BD Microbiology Systems).^18,19^ Cultures that were positive for the growth of acid-fast bacilli underwent confirmation of *M tuberculosis* complex by MPT64/MPB64 antigen detection.^18^ Culture results were classified as contaminated, *M tuberculosis* detected, or no *M tuberculosis* detected. For Ultra testing, 1·4 mL sample reagent was added to 0·7 mL of the resuspended pellet (2:1 dilution) and 2·0 mL of the resulting mixture was added to one Ultra cartridge and tested as described.

Staff performing Ultra and Xpert assays were masked to the results of the other study tests through specimen codes and staffing assignments. Laboratory staff did not have access to participants’ clinical information. Data were captured through dedicated password-protected data entry systems.

The study was reviewed and approved by institutional review or ethics committees at each site. Written informed consent was obtained from all participants.

### Statistical analysis

The reference standard was the sputum 3 culture result, inclusive of results of MGIT and Löwenstein–Jensen medium. If either or both of the reference tests were positive for *M tuberculosis*, then the reference standard was considered positive for *M tuberculosis*. If both were negative for *M tuberculosis*, or if one was negative and the other was contaminated, then the reference standard was considered negative for *M tuberculosis* (figure 1). The comparator test was MGIT culture on the resuspended pellet from sputum 2 (MGIT-Sp2-pellet). Two comparisons were prespecified. For comparison A, the index test was the Ultra result from unprocessed sputum 1 (Ultra-Sp1-raw). For comparison B, the index test was the Ultra result from the resuspended pellet from sputum 2 (Ultra-Sp2-pellet). We also determined the sensitivities of Xpert using unprocessed sputum 1 (Xpert-Sp1-raw) and Löwenstein–Jensen culture using resuspended pellet from sputum 2 (LJ-Sp2-pellet).

Ultra and Xpert results of *M tuberculosis* detected and *M tuberculosis* not detected were classified as determinate. Results of invalid, error, and no result were classified as non-determinate. Cultures with growth of *M tuberculosis* or no growth of *M tuberculosis* were classified as determinate, and contaminated cultures were classified as non-determinate. A participant was classified as smear-positive if any study smear was positive for acid-fast bacilli by smear microscopy or as smear-negative if all study smears were negative for acid-fast bacilli (smear scanty results were considered positive). Sensitivity was defined as the proportion of participants testing positive for *M tuberculosis* with the reference standard who had also tested positive for *M tuberculosis* with the test of interest. Specificity was defined as the proportion of participants testing negative for *M tuberculosis* using the reference standard who also tested negative for *M tuberculosis* using the test of interest.

Participants who provided fewer than three sputum specimens or for whom the reference standard result was non-determinate were excluded from analyses. CIs for simple proportions were calculated using the Wilson score interval.

The primary hypothesis was that the sensitivity of a single Ultra test was non-inferior to that of a single MGIT culture. To assess non-inferiority, the upper limit of the 95% CI of the difference in sensitivity (expressed as comparator sensitivity minus index test sensitivity) was compared with the predefined non-inferiority margin of 5·0 percentage points. Non-inferiority was considered to be achieved if the upper limit of the 95% CI of the sensitivity difference was no greater than the non-inferiority margin. The margin of non-inferiority was selected based on informal clinician input with regard to the potential benefits of a rapid, more easily implemented diagnostic test weighed against potential missed diagnoses. We used Tango’s score method for matched pairs to derive CIs for differences in sensitivity between comparator and index tests.^20^ Participants without a determinate result on both the index and comparator test were omitted from non-inferiority analyses. In an exploratory post-hoc analysis, Ultra semiquantitative trace results were considered negative for *M tuberculosis*. There was no prespecified non-inferiority margin for specificity comparisons.

Sample size calculations were done by Monte Carlo simulation, conservatively assuming a moderate correlation of 0·5 between tests when testing samples from the same participant using two different methods. We generated 10 000 correlated binary data sets for each simulation, using a variety of input test sensitivity parameters. The final sample size was selected to show superiority in 80% or more of the simulated datasets, with parameter estimates of 88% for Ultra sensitivity and of 90% for MGIT sensitivity for case detection and non-inferiority margin of 5%. We calculated that 671 reference standard positive participants were required.

### Role of the funding source

The funder of the study had no role in study design, data collection, data analysis, data interpretation, or writing of the report.

## Results

**Figure 2:**
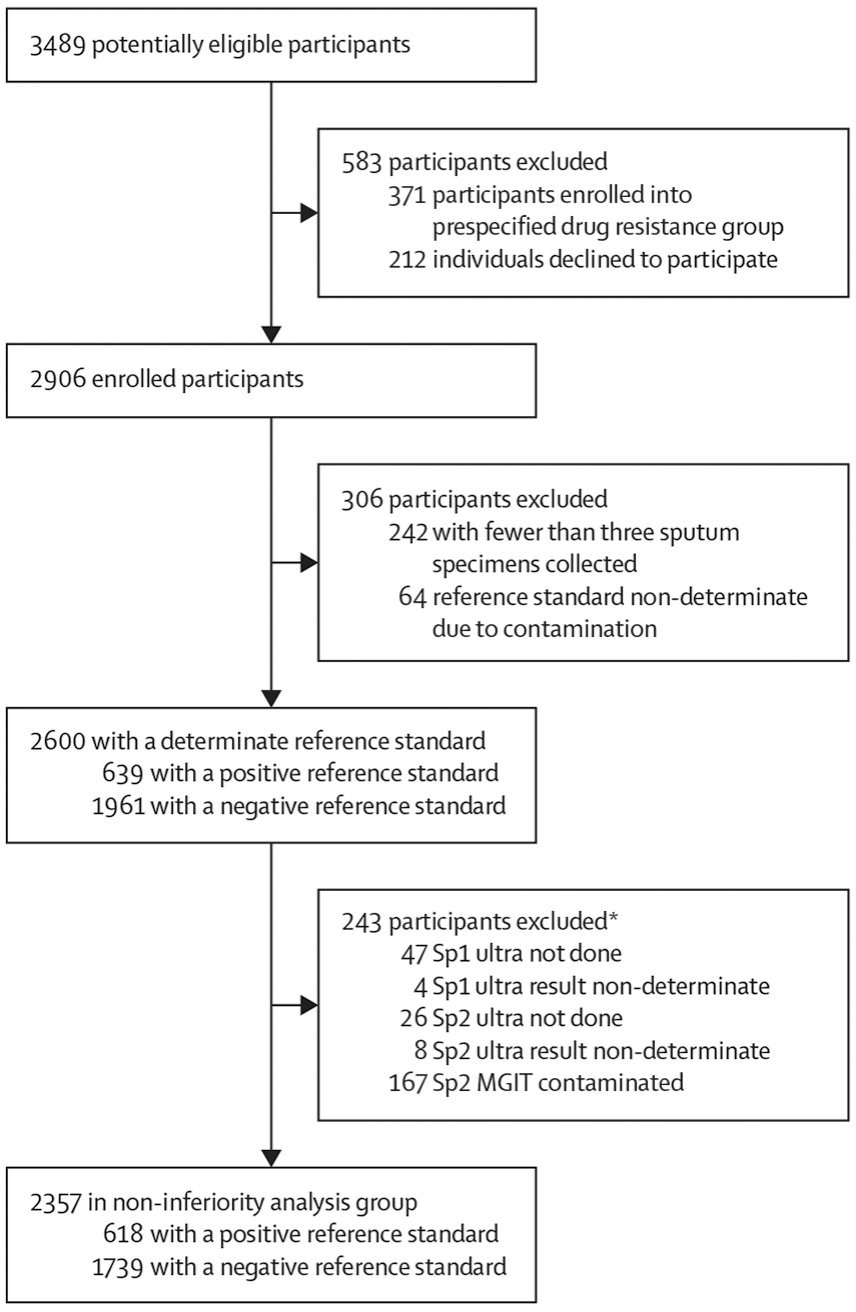
Participant selection. *One participant met more than one exclusion criterion MGIT=mycobacterial growth indicator tube liquid culture.

Between Feb 18, 2016, and Dec 4, 2019, we screened 3489 people for potential enrolment and enrolled 2906 (83%; figure 2). Among enrolled participants, 2600 (89%) were included in analyses. 242 (8%) participants were excluded because fewer than three sputum specimens were collected and 64 (2%) were excluded because their reference standard cultures were contaminated. Among 2600 participants analysed, 2357 (91%) had determinate results for Ultra-Sp1-raw, Ultra-Sp2-pellet, and MGIT-Sp2-pellet and were included in non-inferiority analyses. Among 2357 participants in the non-inferiority analysis population, 877 (37%) were HIV-positive and 525 (22%) reported a history of previous treated tuberculosis (table 1). 618 (26%) participants were positive for *M tuberculosis* by the reference standard, including 477 (77%) of 618 who were positive using sputum smear microscopy.

**Figure 3:**
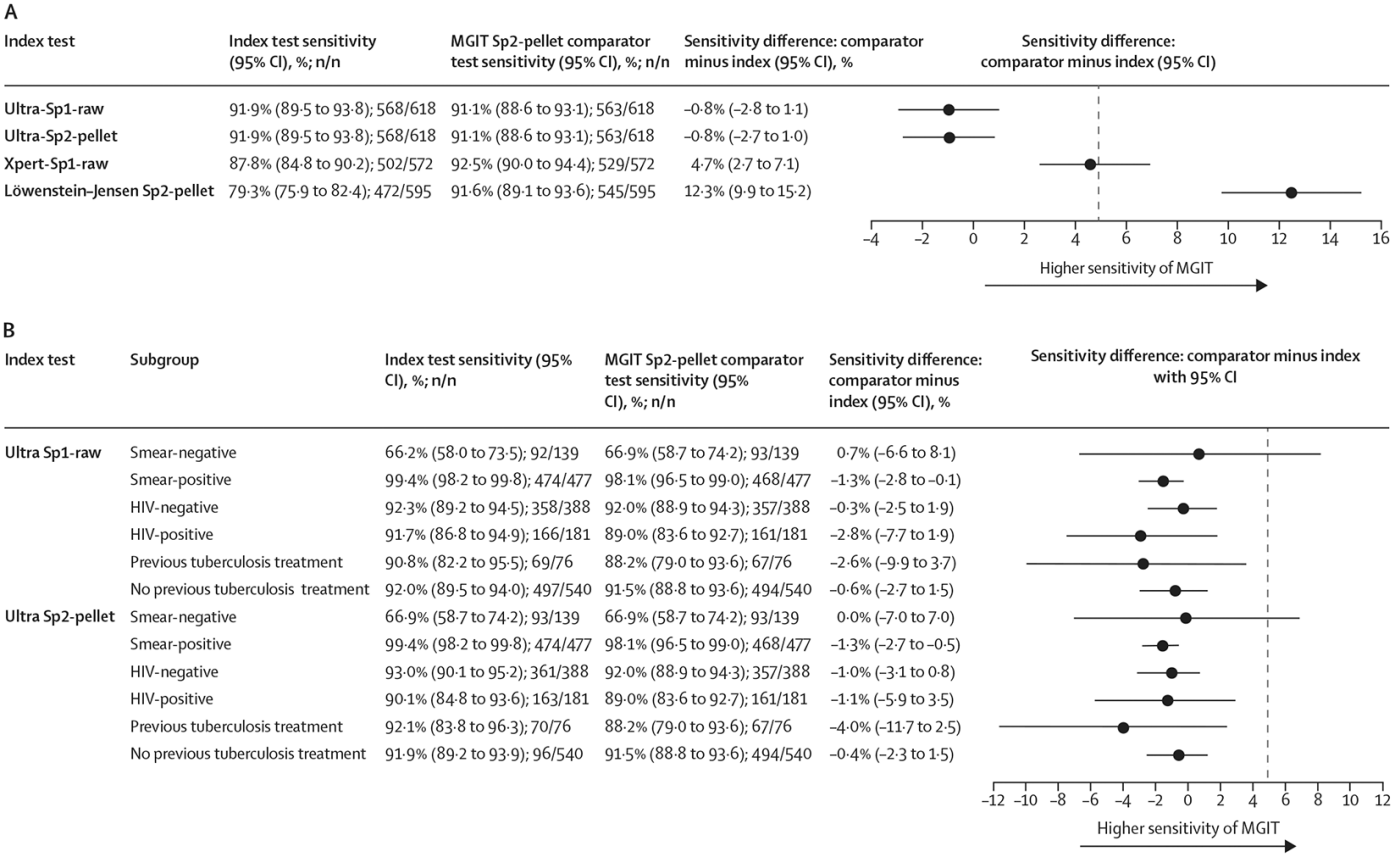
Results of pairwise comparisons of sensitivity between the comparator MGIT culture test and index tests for the non-inferiority analysis population The dotted vertical line represents the margin of non-inferiority (5·0%). (A) Primary analysis. (B) Subgroup analyses. MGIT=mycobacterial growth indicator tube liquid culture.

In the primary analysis, the sensitivity of Ultra performed directly on sputum 1 (Ultra-Sp1-raw) was non-inferior to that of sputum 2 MGIT culture (MGIT 91·1% *vs* Ultra 91·9%; difference −0·8 percentage points; 95% CI −2·8 to 1·1; figure 3A). The sensitivity of Ultra performed on sputum 2 pellet (Ultra-Sp2-pellet) was also non-inferior to that of sputum 2 MGIT (MGIT 91·1% *vs* Ultra 91·9%; difference −0·8 percentage points; 95% CI −2·7 to 1·0). Sensitivity differences between Ultra and MGIT test pairs were also assessed by baseline sputum smear microscopy status, HIV status, and previous tuberculosis treatment status. Sensitivity point estimates were slightly higher for Ultra tests than for MGIT in all subgroups except for sputum smear-negative participants (figure 3B).

In post-hoc analyses in which Ultra semiquantitative results of trace were considered negative for *M tuberculosis*, the sensitivity of Ultra-Sp1-raw (89·2%) remained non-inferior to that of MGIT culture (difference 1·9 percentage points; 95% CI −0·1 to 4·1). The sensitivity of Ultra-Sp2-pellet was not non-inferior to that of MGIT (difference 3·7 percentage points; 1·8 to 5·9; table 2). When the results were further stratified by previous tuberculosis history (table 2), reclassification of trace Ultra results as negative resulted in a decrement in Ultra-Sp2-pellet sensitivity from 92·1% (70 of 76; 83·8 to 96·3) to 77·6% (59 of 76; 67·1 to 85·5), whereas Ultra-Sp1-raw sensitivity was less affected.

Compared with the sensitivity of sputum 2 MGIT, the sensitivity of Xpert-Sp1-raw was not non-inferior (92·5% [529 of 572; 95% CI 90·0 to 94·4] for MGIT *vs* 87·8% [502 of 572; 84·8 to 90·2] for Xpert-Sp1-raw; difference 4·7 percentage points; 2·7 to 7·1) and the sensitivity of sputum 2 Löwenstein–Jensen culture was inferior (91·6% [545 of 595; 89·1 to 93·6] for MGIT *vs* 79·3% [472 of 595; 75·9 to 82·4] for Löwenstein–Jensen; difference 12·3 percentage points; 9·9 to 15·2).

In the non-inferiority analysis group, MGIT specificity was 97·1% (1688 of 1739; 95% CI 96·2 to 97·8). Ultra-Sp1-raw specificity was 94·4% (1642 of 1739; 93·2 to 95·4) with a difference of 2·7 percentage points (1·7 to 3·8) versus MGIT. Ultra-Sp2-pellet specificity was 91·0% (1583 of 1739; 89·6 to 92·3), with a difference of 6·1 percentage points (4·9 to 7·5). In post-hoc analyses in which Ultra trace results were considered negative for *M tuberculosis*, Ultra-Sp1-raw specificity was 95·7% (1664/1739; 94·6 to 96·6) with a difference of 1·4 percentage points (0·5 to 2·4) versus MGIT, and Ultra-Sp2-pellet specificity was 96·2% (1672 of 1739; 95·1 to 97·0) with a difference 1·0 percentage point (0·1 to 1·9) versus MGIT (table 2).

The non-inferiority analysis required determinate results for both index and comparator tests in a pair and, therefore, excluded some determinate results. Accordingly, we calculated the sensitivity and specificity for each test type considered independently (appendix p 2). The sensitivity was 91·5% (581 of 635; 95% CI 89·1 to 93·4) for Ultra-Sp1-raw, 91·5% (581 of 635; 89·1 to 93·4) for Ultra-Sp2-pellet, and 90·9% (568 of 625; 88·4 to 92·9) for MGIT-Sp2-pellet. The specificity was 94·3% (1804 of 1914; 93·1 to 95·2) for Ultra-Sp1-raw, 91·1% (1759 of 1931; 89·7 to 92·3) for Ultra-Sp2-pellet, and 97·2% (1758 of 1808; 96·4 to 97·9) for MGIT-Sp2-pellet. Among tests initiated, a final result of non-determinate occurred in 0·2% (four of 2553) of Ultra-Sp1-raw test, 0·3% (eight of 2574) of Ultra-Sp2-pellet test, and 6·4% (167/2600) of MGIT-Sp2-pellet tests. Contamination rates for MGIT and Löwenstein–Jensen were between 6·1% and 9·9% (appendix p 3).

## Discussion

The sensitivity of one Ultra test was non-inferior to that of one MGIT liquid culture for detection of *M tuberculosis* in expectorated sputum specimens. Non-inferiority was demonstrated when MGIT and Ultra tests were performed on the same resuspended processed sputum pellet and when Ultra tests were performed directly on a different raw, unprocessed sputum specimen. In the primary analysis, there was no evidence that Ultra test sensitivity was less than from that of MGIT.

Results were consistent when stratified by participant sputum smear microscopy status and by HIV status. Ultra sensitivity was lower in participants who were smear-negative versus smear-positive, supporting an influence of airway bacillary burden on test sensitivity, as has been previously reported.^6,7^ Our results extend this observation to MGIT culture, for which sensitivity was substantially lower among participants who were smear-negative compared with those who were smear-positive. MGIT and Ultra sensitivities were slightly lower in participants with HIV versus those without HIV, a finding that might also reflect differences in airway bacillary burden.^21,22^ The sensitivities of MGIT-Sp2-pellet, Ultra-Sp2-pellet, and Ultra-Sp1-raw were mostly similar to each other across clinically important subgroups of participants living with HIV and participants with low bacillary burden in sputum. These findings provide reassurance that Ultra-based diagnostic testing algorithms maintain sensitivity in settings where HIV is prevalent.

We selected a non-inferiority study design due to the high intrinsic sensitivity of the comparator MGIT culture, such that a superiority trial would not be practicable, and because Ultra has attributes that might be sufficiently favourable to outweigh a potential small reduction in sensitivity. To our knowledge this is the first study to use a non-inferiority design in tuberculosis diagnostics, but might become increasingly necessary given the emergence of other point-of-care nucleic acid amplification tests for tuberculosis diagnosis. We selected the non-inferiority margin based on informal input from tuberculosis clinicians, although a Delphi-type approach would have been more rigorous. Nonetheless, our conclusion of non-inferiority of sensitivity is not highly influenced by the absolute margin.

The proportion of participants with a determinate test result was higher for Ultra than for MGIT, which has important practical implications, especially in settings with low resources for testing. Under a hypothetical testing scenario, in which only one sputum specimen was tested, the use of Ultra would be expected to detect slightly more pulmonary tuberculosis cases than would the use of MGIT culture given Ultra’s comparable sensitivity and higher proportion of determinate results. Non-determinate sputum 2 MGIT results were likely due to contamination rates of 6·6%. A contamination rate of 5–8% is considered the benchmark rate that balances the competing risks of false-negative cultures due to loss of mycobacterial viability during sputum decontamination procedures with overgrowth of upper airway flora due to insufficient sputum decontamination.^17^

Specificity point estimates for Ultra and Xpert were lower in our study than in some other recent studies.^6,7^ A contributing factor to the lower specificity point estimates in our study was the analysis approach, in which results of only one sputum specimen (ie, sputum 3) were considered for reference standard classification of each participant. We used this approach to designate the sputum 2 MGIT test as the comparator test, given that our overall study hypothesis focused on test sensitivity and to maintain an overall testing approach in which no more than three sputum specimens were required from each participant. A reference standard that includes results of two or more sputum specimens reduces the effect of sputum-to-sputum bacillary burden heterogeneity on participant classification since isolation of *M tuberculosis* from any of the corresponding cultures would classify the participant as reference-standard positive. This reference standard also reduces the number of participants excluded from the analysis due to a non-determinate reference standard. In the current study, the sputum 2 MGIT specificity of 96·6% provides some insight into the effect of specimen-to-specimen heterogeneity, since 51 (2·9%) of 1739 specimens that were negative for *M tuberculosis* by the culture-based sputum 3 reference standard were positive for *M tuberculosis* by sputum 2 MGIT culture.

Our results extend previous observations that Ultra detects *M tuberculosis* in some specimens from which *M tuberculosis* cannot be cultivated using routine methods.^6,23-28^ The biological underpinnings for such discordant results have not been identified; however, work in this area could improve understanding of the pathobiology of tuberculosis when considered as a disease spectrum. When tuberculosis is viewed as a dichotomous condition, possible explanations for discordant results between Ultra and culture include false-positive Ultra results due to dead bacilli or contamination and false-negative cultures.^26^ MGIT culture is an imperfect reference standard that can give false-negative results due to the killing of *M tuberculosis* during the sputum decontamination process or the presence of differentially cultivatable bacilli that do not grow in routine culture.^14,15^ These scenarios would most probably occur with a low, rather than high, sputum bacillary burden. Consistent with this, Ultra-positive and culture-negative rates as high as 48–65% have been observed in high prevalence settings for tuberculosis where people were tested regardless of symptoms, whereas concordance between Ultra and culture has been substantially higher in people with pulmonary tuberculosis symptoms, as in our study.^26,27,29,30^ As expected, the designation of Ultra trace results as negative increased specificity but reduced sensitivity, although Ultra-Sp2-raw sensitivity remained non-inferior to that of the MGIT comparator. From a tuberculosis programme perspective, this trade-off between sensitivity and specificity is weighted against factors, including the prevalence of tuberculosis, the prevalence of comorbidities that affect tuberculosis morbidity and mortality, and the resources for close follow-up of people with Ultra trace results who are on tuberculosis treatment.^31^

A key study strength is the ability to compare the sensitivities of Ultra and MGIT performed on aliquots of the same specimen (ie, resuspended pellet from sputum 2). The diversity of study settings, inclusion of people living with and without HIV, and heterogeneity in sputum bacillary burden contribute to the generalisability of study findings. A study limitation is that 8% of enrolled participants were excluded from analyses because fewer than three sputum specimens were obtained. This analysis approach could have led to selection bias if people with paucibacillary disease were less likely to produce three sputum specimens. The Ultra package insert recommends using a 3:1 ratio of sample reagent to specimen (volume) when testing resuspended processed sputum pellets. This is a convenience recommendation to help ensure an adequate specimen volume for Ultra testing in the event that the residual pellet volume is low. We used a 2:1 ratio of sample reagent to specimen pellet volume based on previous work showing superior sensitivity versus a 3:1 dilution.^32^ Our study was not designed to identify the optimal number of sputum specimens per person that should be tested using Ultra to optimise the diagnostic yield of a clinical testing algorithm. We acknowledge that tuberculosis diagnostic guidelines recommend testing more than one sputum sample and that Ultra testing on processed sputum pellets typically would not be done at point-of-care. With respect to study design, we used the MGIT culture method as a comparator (specimen 2) and a reference standard (specimen 3) and we mitigated potential bias by not including specimen 2 MGIT results in the reference standard.

Our findings provide new, clinically relevant information about the head-to-head comparative diagnostic sensitivity of Ultra and MGIT culture for the detection of *M tuberculosis* in sputum specimens. Results should help policy makers and tuberculosis programmes calculate the relative merits and drawbacks of diagnostic testing strategies that incorporate these assays. Highly sensitive, rapid molecular approaches for tuberculosis case detection combined with advances in genotypic approaches to drug resistance detection have the potential to replace culture.

## Supplementary Material

1

## Figures and Tables

**Table 1:** Characteristics of the study population

	Eligible population (n=2906)	Determinate reference standard population (n=2600)	Non-inferiority analysis population (n=2357)
Median age (IQR), years	37 (19)	37 (19)	38 (20)
Sex			
Female	1213 (42%)	1094 (42%)	984 (42%)
Male	1692 (58%)	1506 (58%)	1373 (58%)
Not reported	1 (0%)	0 (0%)	0 (0%)
Enrolment country			
Belarus	52 (2%)	52 (2%)	50 (2%)
Brazil	174 (6%)	160 (6%)	152 (6%)
China	37 (1%)	35 (1%)	35 (1%)
Georgia	375 (13%)	369 (14%)	341 (14%)
India	247 (8%)	190 (7%)	176 (7%)
Kenya	494 (17%)	416 (16%)	391 (17%)
South Africa	901 (31%)	793 (31%)	716 (30%)
Uganda	626 (22%)	585 (23%)	496 (21%)
HIV status			
HIV-positive	1123 (39%)	989 (38%)	877 (37%)
HIV-negative	1654 (57%)	1521 (59%)	1397 (59%)
Unknown HIV status	129 (4%)	90 (3%)	83 (4%)
History of previous treated tuberculosis			
Yes	629 (22%)	573 (22%)	525 (22%)
No	2272 (78%)	2024 (78%)	1829 (78%)
Unknown	5 (0%)	3 (0%)	3 (0%)
MRS results			
MRS-positive for *Mycobacterium tuberculosis*	639 (22%)	639 (25%)	618 (26%)
Any smear-positive	487 (17%)	487 (19%)	477 (20%)
No smear-positive	150 (5%)	150 (6%)	139 (6%)
MRS-negative for *M tuberculosis*	1961 (67%)	1961 (75%)	1739 (74%)
Non-determinate due to contamination or third specimen not collected	306 (11%)	NA	NA

MRS=microbiological reference standard. NA=not applicable.

**Table 2:** Sensitivity and specificity of Ultra *vs* MGIT, by interpretation of trace Ultra results and history of prior treated tuberculosis

	Non-inferiority analysis group			No previous history of tuberculosis			Previously treated tuberculosis		
	Index test sensitivity	MGIT-Sp2-pellet comparator test sensitivity	Sensitivity difference: comparator minus index	Index test sensitivity	MGIT-Sp2-pellet comparator test sensitivity	Sensitivity difference: comparator minus index	Index test sensitivity	MGIT-Sp2-pellet comparator test sensitivity	Sensitivity difference: comparator minus index
**Sensitivity**									
Ultra-Sp1-raw, trace as *Mycobacterium tuberculosis*-positive	91·9% (89·5 to 93·8); 568/618	91·1% (88·6 to 93·1); 563/618	−0·8% (−2·8 to 1·1)	92·0% (89·5 to 94·0); 497/540	91·5% (88·8 to 93·6); 494/540	−0·6% (−2·7 to 1·5)	90·8% (82·2 to 95·5); 69/76	88·2% (79·0 to 93·6); 67/76	−2·6% (−9·9 to 3·7)
Ultra-Sp1-raw, trace as *M tuberculosis*-negative	89·2% (86·5 to 91·4); 551/618	..	1·9% (−0·1 to 4·1)	89·3% (86·4 to 91·6); 482/540	..	2·2% (0·1 to 4·6)	88·2% (79·0 to 93·6); 67/76	..	0·0% (−7·7 to 7·7)
Ultra-Sp2-pellet, trace as *M tuberculosis*-positive	91·9% (89·5 to 93·8); 568/618	..	−0·8% (−2·7 to 1·0)	91·9% (89·2 to 93·9); 496/540	..	−0·4% (−2·3 to 1·5)	92·1% (83·8 to 96·3); 70/76	..	−4·0% (−11·7 to 2·5)
Ultra-Sp2-pellet, trace as *M tuberculosis*-negative	87·4% (84·5 to 90·0); 540/618	..	3·7% (1·8 to 5·9)	88·7% (85·8 to 91·1); 479/540	..	2·8% (0·8 to 5·0)	77·6% (67·1 to 85·5); 59/76	..	10·5% (3·0 to 20·0)
**Specificity**									
Ultra-Sp1-raw, trace as *M tuberculosis*-positive	94·4% (93·2 to 95·4); 1642/1739	97·1% (96·2 to 97·8); 1688/1739	2·7% (1·7 to 3·8)	95·0% (93·7 to 96·1); 1225/1289	96·7% (95·6 to 97·6); 1247/1289	1·7% (0·6 to 2·9)	92·7% (89·9 to 94·7); 416/449	98·2% (96·5 to 99·1); 441/449	5·6% (3·2 to 8·4)
Ultra-Sp1-raw, trace as *M tuberculosis*-negative	95·7% (94·6 to 96·6); 1664/1739	..	1·4% (0·5 to 2·4)	96·8% (95·7 to 97·7); 1248/1289	..	−0·1% (−1·1 to 0·9)	95·1% (92·7 to 96·7); 427/449	..	3·1% (1·1 to 5·5)
Ultra-Sp2-pellet, trace as *M tuberculosis*-positive	91·0% (89·6 to 92·3); 1583/1739	..	6·1% (4·9 to 7·5)	91·3% (89·7 to 92·7); 1177/1289	..	5·4% (4·1 to 7·0)	90·2% (87·1 to 92·6); 405/449	..	8·0% (5·4 to 11·1)
Ultra-Sp2-pellet, trace as *M tuberculosis*-negative	96·2% (95·1 to 97·0); 1672/1739	..	1·0% (0·1 to 1·9)	96·4% (95·3 to 97·3); 1243/1289	..	0·3% (−0·7 to 1·3)	95·3% (93·0 to 96·9); 428/449	..	2·9% (0·8 to 5·3)

Data are in % (95% CI); n/N or % (95% CI). MGIT=mycobacterial growth indicator tube liquid culture.

## Data Availability

De-identified participant data can be accessed on reasonable request to the corresponding author.

## Research in context

### Evidence before this study

Liquid culture is considered the most sensitive routinely available test for the detection of *Mycobacterium tuberculosis* in clinical specimens. Xpert MTB/RIF Ultra (Ultra) has also been shown to have high diagnostic sensitivity; however, almost all published studies report the sensitivity of Ultra testing with a single sputum specimen using a microbiological reference standard comprising liquid cultures of multiple sputum specimens. To ascertain whether head-to-head comparisons of the diagnostic sensitivity of a single liquid culture versus that of a single Ultra test had been done, we searched PubMed with the terms ((pulmonary tuberculosis or pulmonary TB) AND Ultra AND (MGIT or liquid culture) AND comparative sensitivity or head-to-head)) for publications between Jan 1, 2008, and Feb 8, 2023. We identified and reviewed 13 publications. No publication described head-to-head comparisons of Ultra and liquid culture in adults who are symptomatic for tuberculosis.

### Added value of this study

To our knowledge, we present the first evaluation of the comparative diagnostic sensitivities of Ultra and mycobacterial growth indicator tube (MGIT) culture for the detection of *M tuberculosis* in sputum specimens. Using a culture-based reference standard, we made two comparisons—namely, Ultra versus MGIT done on the same processed resuspended sputum pellet and Ultra done directly on one unprocessed sputum specimen versus MGIT done on the processed sputum pellet from a different sputum specimen. Ultra was as sensitive as MGIT in both of these comparisons.

### Implications of all the available evidence

Our results showing that Ultra has a sensitivity that is similar to that of MGIT are novel. This information will help policy makers and tuberculosis programmes calculate the relative merits and drawbacks of diagnostic testing strategies that incorporate these assays. Our findings support a transition away from culture-based tuberculosis diagnostic algorithms and towards molecular approaches for pulmonary tuberculosis case detection. The crucial next step to this transition requires biological and technical advances that yield comprehensive drug susceptibility information from molecular tests performed directly on sputum.
