# Supplementary material for: Xpert MTB/RIF Ultra versus mycobacterial growth indicator tube liquid culture for detection of Mycobacterium tuberculosis in symptomatic adults: a diagnostic accuracy study
Source: Lancet Microbe. Author manuscript; Available in PMC 2026 Jan 14. (PMC12802551; doi:10.1016/S2666-5247(24)00001-6)
Supplement: 1 [file NIHMS1999945-supplement-1.pdf]

THE LANCET
Microbe

Supplementary appendix

This appendix formed part of the original submission and has been peer reviewed.
We post it as supplied by the authors.

Supplement to: Xie YL, Eichberg C, Hapeela N, et al. Xpert MTB/RIF Ultra versus mycobacterial growth indicator tube liquid culture for detection of *Mycobacterium tuberculosis* in symptomatic adults: a diagnostic accuracy study. *Lancet Microbe* 2024. https://doi.org/10.1016/S2666-5247(24)00001-6

Supplementary Material

Diagnostic sensitivity of Xpert MTB/RIF Ultra is non-inferior to liquid culture for detection of *Mycobacterium tuberculosis*

Yingda L. Xie, Christie Eichberg, Nchimunya Hapeela, Elizabeth Nakabugo, Irene Anyango, Kiranjot Arora, Jeffrey Korte, Ronald Odero, Judi Van Heerden, Widaad Zemanay, Samuel Kennedy, Pamela Nabeta, Mahmud Hanif, Camilla Rodrigues, Alena Skrahina, Wendy Stevens, Reynaldo Dietze, Xin Liu, Jerrold J. Ellner, David Alland, Moses L. Joloba, Samuel G. Schumacher, Kimberly D. McCarthy, Lydia Nakiyingi, Susan E. Dorman

Table of Contents

Page 2
Supplementary Table 1.
Test sensitivity and specificity for participants in the determinate reference standard group.

Page 3
Supplementary Table 2.
Culture contamination rates among 2906 enrolled participants.

Page 4
Supplementary Table 3.
Test results for the group of 64 participants with a contaminated reference standard.

Page 5
Study Protocol

Supplementary Table 1. Test sensitivity and specificity estimates for participants in the determinate reference standard group

A) SENSITIVITY

Index test	Sensitivity n/n % (95% CI)	Index test negative for MTB, (n)	Other index test results (not included in sensitivity calculation)	
			Index test with nondeterminate result (n)	Index test not done (n)
Ultra-Sp1-raw	581/635<br>91.5%<br>(89.1%, 93.4%)	54	0	4
Ultra Sp2-pellet	581/635<br>91.5%<br>(89.1%, 93.4%)	54	1	3
MGIT Sp2-pellet	568/625<br>90.9%<br>(88.4%, 92.9%)	57	14[a]	0
Xpert-Sp1-raw	515/592<br>87.0%<br>(84.0%, 89.5%)	77	0	47[b]
LJ-Sp2-pellet	482/614<br>78.5%<br>(75.1%, 81.6%)	132	22[a]	3

B) SPECIFICITY

Index test	Specificity n/n % (95% CI)	Index test positive for MTB, (n)	Other index test results (not included in specificity calculation)	
			Index test with nondeterminate result (n)	Index test not done (n)
Ultra-Sp1-raw	1804/1914<br>94.3%<br>(93.1%, 95.2%)	110	4	43
Ultra Sp2-pellet	1759/1931<br>91.1%<br>(89.7%, 92.3%)	172	7	23
MGIT Sp2-pellet	1758/1808<br>97.2%<br>(96.4%, 97.9%)	50	153[c]	0
Xpert-Sp1-raw	1572/1628<br>96.6%<br>(95.6%, 97.3%)	56	0	333[d]
LJ-Sp2-pellet	1785/1815<br>98.3%<br>(97.7%, 98.8%)	30	137[a]	9

Abbreviations: CI, confidence interval; MTB, *Mycobacterium tuberculosis*; MGIT, Mycobacterial Growth Indicator Tube liquid culture
[a]contaminated cultures; [b]46 from South Africa site where Xpert MTB/RIF version not available; [c]contaminated cultures; [d]331 from South Africa site where Xpert MTB/RIF version not available.

**Supplementary Table 2. Culture contamination rates among 2906 enrolled participants**		
	% contaminated	n/n
MGIT-Sp2-pellet	6.6%	183/2786 [a]
LJ-Sp2-pellet	6.1%	169/2786 [a]
MGIT-Sp3-pellet	9.9%	264/2676 [b]
LJ-Sp2-pellet	8.3%	220/2661 [c]

Abbreviations: MGIT, Mycobacterial growth index tube; LJ, Lowenstein Jensen
[a] 120 cultures not done (sputum not collected)
[b] 230 cultures not done (sputum not collected)
[c] 245 cultures not done (sputum not collected)

**Supplementary Table 3. Index and comparator test results for the group of n=64 participants with a contaminated reference standard**				
Index test	# positive for MTB among tests performed (%)	# negative for MTB among tests performed (%)	# nondeterminate among tests performed (%)	# not done
Ultra-Sp1-raw	4 (6%)	60 (94%)	0 (0%)	0
Ultra-Sp2-pellet	6 (9%)	58 (91%)	0 (0%)	0
MGIT-Sp2-pellet	3 (5%)	53 (83%)	8 (13%)	0
Xpert-Sp1-raw	2 (5%)	41 (95%)	0 (0%)	21[a]
LJ-Sp2-pellet	3 (5%)	58 (91%)	3 (5%)	0

Abbreviations: MTB, Mycobacterium tuberculosis; MGIT, Mycobacterial Growth Indicator Tube liquid culture; LJ, Lowenstein-Jensen solid culture
[a]21 participants from South Africa site where Xpert MTB/RIF version not available

Multicenter Study of the Accuracy and Feasibility of the Xpert Ultra Test

DMID Protocol Number: 15-0029

Sponsored by:
National Institute of Allergy and Infectious Diseases (NIAID)

DMID Funding Mechanism: R01 AI129411

Principal Investigators: Susan Dorman, MD; Claudia Denkinger, MD; David Alland, MD

DMID Project Officer: Karen Lacourciere, PhD

DMID Clinical Project Manager: Tena Knudsen, RN

Version Number: 9.0

Day Month Year: 16 Apr 2020

Statement of Compliance

The study will be conducted in compliance with the protocol, and carried out in accordance with Good Clinical Practice (GCP) as required by the following:

- U.S. Code of Federal Regulations applicable to clinical studies (45 CFR 46)
- ICH GCP E6
- Completion of Human Subjects Protection Training
- the NIH Clinical Terms of Award

SIGNATURE PAGE

The signature below constitutes the approval of this protocol and the attachments, and provides the necessary assurances that this study will be conducted according to all stipulations of the protocol, including all statements regarding confidentiality, and according to local legal and regulatory requirements and applicable US federal regulations and ICH guidelines.

Site Investigator:*

Signed: ______________________________ Date: ____________

Name
Title

** The protocol should be signed by the local investigator who is responsible for the study implementation at his/her specific site.*

Statement of Compliance i
Signature Page ii
List of Abbreviations v
Protocol Summary vi
Schematic of Study Design viii

1 Key Roles 1
2 Background Information and Scientific Rationale 5
2.1 Background Information 5
2.2 Rationale 7
2.3 Potential Risks and Benefits 8
2.3.1 Potential Risks 8
2.3.2 Known Potential Benefits 8
3 Objectives 10
4 Study Design 11
4.1 Study Endpoints 11
4.1.1 Primary 11
4.1.2 Secondary 11
4.1.3 Exploratory 12
4.2 Timeline (projected) 12
4.3 Blinding 13
5 SUBJECT SELECTION AND ENROLLMENT 14
5.1 Selection of the Study Population and Recruitment 14
5.2 Inclusion/Exclusion Criteria 14
5.2.1 Inclusion Criteria 14
5.2.2 Exclusion Criteria 14
5.3 Early Withdrawals 14
5.4 Selection of Participants for Follow-up over 12 Months 15
5.5 Voluntary Withdrawal by a Participant 15
5.6 Termination of the Study 15
6 Study Procedures 17
6.1 Screening Procedures 17
6.2 Enrollment/Baseline (Day 1 and Day 2) 17
6.2.1 Clinical 17
6.2.2 Laboratory testing of sputa S1, S2, S3, S4 18
6.3 Follow-up Visits 19
6.3.1 Day 7 Follow-Up Visit 19
6.3.2 Month 2 Follow-Up Visit 19
6.3.3 Month 6 Follow-Up Visit (in-person) 20
6.3.4 Month 12 Follow-Up Visit (in-person) 21
6.4 Specimen Handling, Storage, and Shipping 22
6.4.1 Xpert MTB/RIF Ultra cartridges for which the test result was ‘MTB detected’ 22
6.4.2 Sputum for *M. tuberculosis* gene expression studies 22
6.4.3 Blood for human gene expression studies 22
6.4.4 Urine and blood (serum) for investigational *M. tuberculosis* antigen studies 23

6.4.5 Incubation of MGIT cultures with growth supplement 23
6.5 Participant Compensation 23
6.6 Collection of Cost Data 23
7 Investigational products 24
7.1 Investigational Products Description 24
7.2 Acquisition 24
7.3 Storage 24
7.4 Test Handling and Performance 24
7.5 Export and Import Permits 24
7.6 Quality Control Check for Incoming Shipments 24
8 Safety assessment and reporting 25
9 Clinical Monitoring 26
9.1 Site Monitoring Plan 26
10 Statistical Considerations 27
10.1 Study Hypothesis 27
10.2 Analysis Plans 27
10.2.1 Primary Endpoint and Hypothesis 27
10.2.2 Secondary Endpoints 28
10.3 Sample Size Considerations 29
11 Quality Control and Quality Assurance 30
12 Ethics/Protection of Human Subjects 31
12.1 Ethical Standard 31
12.2 Institutional Review Board 31
12.3 Informed Consent Process 31
12.4 Subject Confidentiality 31
13 Data MANAGEMENT 33
13.1 Data Management Responsibilities 33
13.2 Data Capture Methods 33
13.3 Types of Data 33
13.4 Study Records Retention 33
13.5 Protocol Deviations 34
14 Literature References 35

SUPPLEMENTS/APPENDICES

A: Study Schedule 37

List of Abbreviations

AFB	Acid-fast bacilli
CDRC	Clinical Diagnostics Research Consortium
CRF	Case Report Form
DMID	Division of Microbiology and Infectious Diseases
DNA	Deoxyribonucleic acid
DST	Drug Susceptibility Testing
FIND	Foundation for Innovative New Diagnostics
FWA	Federal Wide Assurance
GCP	Good Clinical Practice
HIV	Human immunodeficiency virus
ICH	International Conference on Harmonisation
IEC	Independent or Institutional Ethics Committee
IRB	Institutional Review Board
LJ	Lowenstein-Jensen (mycobacterial culture medium)
MUSC	Medical University of South Carolina
MGIT	Mycobacterial Growth Indicator Tube
MTB	*Mycobacterium tuberculosis*
NAAT	Nucleic Acid Amplification Tests
NALC-NaOH	N-acetyl-L-cysteine-sodium hydroxide
NIAID	National Institute of Allergy and Infectious Diseases
NIH	National Institutes of Health, DHHS
OHRP	Office for Human Research Protections, DHHS
PCR	Polymerase chain reaction
PI	Principal Investigator
RIF	Rifampin
TB	Tuberculosis
US	United States
WHO	World Health Organization

PROTOCOL SUMMARY

Title: Multicenter Study of the Accuracy and Feasibility of the Xpert Ultra Test

Population: Adults with signs/symptoms of pulmonary tuberculosis. 880 participants were enrolled in the initial phase between February 2016 and September 2016, and an additional 1200 participants will be enrolled in the expansion phase.

Number of Sites: This study will be undertaken by the TB Clinical Diagnostics Research Consortium (CDRC). Medical University of South Carolina will serve as the overall Coordinating Center. In the initial phase, participant enrollment occurred at five implementing sites (Kisumu, Kenya; Kampala, Uganda; Cape Town, South Africa; Zhengzhou, China; Vitoria, Brazil). For the expansion phase, participant enrollment will occur at three of these sites, namely Kisumu, Kenya; Kampala, Uganda; and Cape Town, South Africa. Centralized data management will be conducted at the Data Coordinating Center housed at Rutgers New Jersey Medical School.

Design: Mainly cross-sectional; certain participants will have longitudinal follow-up

Study Duration: Approximately 48 months

Subject Duration: Approximately two months for most participants; a subset of participants will be followed for approximately 12 months.

Objectives:

Primary:

- To estimate and compare the sensitivity of a single Xpert MTB/RIF Ultra test vs. that of a single MGIT liquid culture for detection of culture-positive pulmonary TB in new TB suspects
- To compare Xpert MTB/RIF Ultra accuracy estimates using revised vs. original assay definition file cut-offs

Secondary:

- To estimate and compare sensitivity and specificity of the Xpert MTB/RIF Ultra test vs. standard Xpert MTB/RIF for detection of rifampin resistance
- To estimate and compare the sensitivity and specificity of the Xpert MTB/RIF Ultra test vs. standard Xpert MTB/RIF for detection of culture-positive pulmonary TB in new TB suspects overall, by sputum smear microscopy status, and by HIV status
- To estimate and compare the specificity of two Xpert MTB/RIF Ultra test vs. that of a single MGIT liquid culture for detection of culture-positive pulmonary TB in new TB suspects
- To estimate and compare the sensitivity and specificity of the Xpert MTB/RIF Ultra test vs. standard Xpert MTB/RIF for detection of rifampin resistance

- To determine Xpert MTB/RIF Ultra test operating characteristics including proportion of tests with a non-determinate result
- To describe current routine clinical care practices regarding initiation of TB treatment
- To model the potential health impact and cost-effectiveness of TB diagnosis using the Xpert MTB/RIF Ultra
- To estimate the negative predictive value of Xpert MTB/RIF Ultra

Exploratory

- To explore the relationship between Xpert MTB/RIF Ultra positivity and clinical, and microbiological markers of TB disease activity in participants whose enrollment cultures are negative for *M. tuberculosis*

Schematic of Study Design for the Expansion Phase:

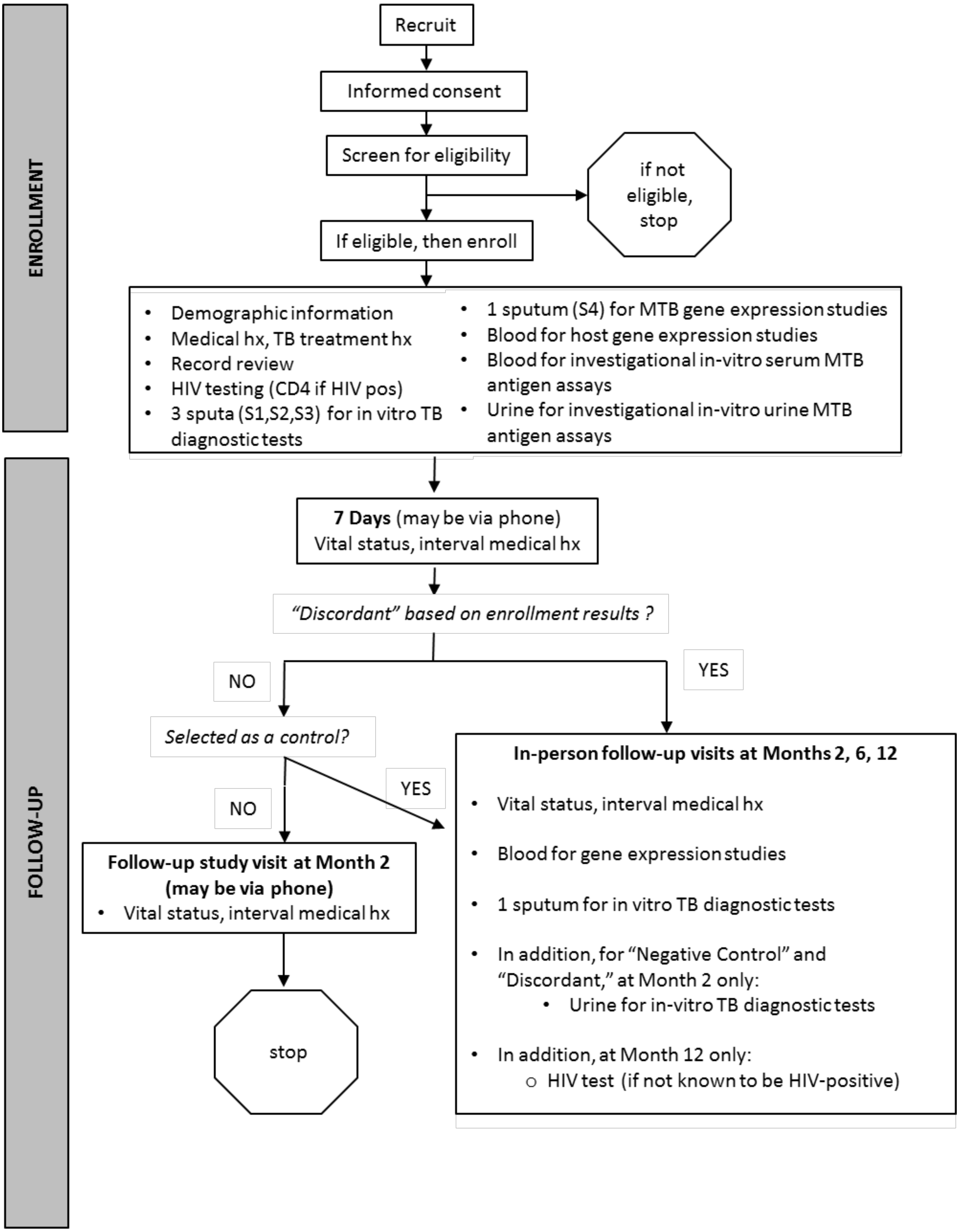

1 KEY ROLES

Individuals:

DMID Project Officer: Karen Lacourciere, PhD; Email lacourcierek@niaid.nih.gov

DMID Clinical Project Manager: Tena Knudsen, RN; Email knudsent@mail.nih.gov

DMID Medical Officer: Mamodikoe Makhene, MD; MMakhene@niaid.nih.gov

Principal Investigators:
Susan E. Dorman, MD
Medical University of South Carolina, 135 Rutledge Ave., Charleston, SC, USA 29425
Email: dorman@musc.edu Tel: 843-792-4541

Claudia Denkinger, MD
Foundation for Innovative New Diagnostics, 16, Ave due Bude, 1202 Geneva, Switzerland
Email: Claudia.Denkinger@finddx.org Tel: +41 022 749 29 31

David Alland, MD
Rutgers New Jersey Medical School,185 South Orange Ave., Newark, New Jersey, USA 07103
Email: allandda@njms.rutgers.edu Tel: 973-972-2179

Sub-Investigators:
Dr. Lydia Nakiyingi
Infectious Diseases Institute-Makerere University, Mulago Hospital Complex, Kampala, Uganda
Email: lnakiyingi@idi.co.ug Tel: +256 772 468 045

Yukari Manabe, MD
Infectious Diseases Institute-Makerere University
Johns Hopkins University, 1830 East Monument St., 4th floor, Baltimore, Maryland, USA 21231
Email: ymanabe@jhmi.edu Tel: 410-955-8571

Dr. Jerrold Ellner
Rutgers New Jersey Medical School, 185 South Orange Ave., Newark, New Jersey USA 07103
Email: ellnerjj@newark.rutgers.edu Tel: 973-972-7259

Carlos Acuna-Villaorduna, MD
Boston Medical Center, 801 Massachusetts Avenue, Boston, MA, USA 02118
Email: carlos.acuna-villaorduna@bmc.org Tel: 617-414-4290

Dr. Chad Centner
Division of Medical Microbiology, 5th floor, Falmouth Building, Health Sciences Faculty
University of Cape Town Anzio Road, Observatory, 7925, Cape Town, South Africa
Email: chadc@nis.za Tel: +27 21 406 6287

Kim McCarthy
CDC-Kenya, Kenya Medical Research Institute / Division of Global HIV and TB Kisumu, Kenya
Email: KMcCarthy3@cdc.gov Tel: +254 724 255 639

Dr. Yingda Xie, MD
Rutgers New Jersey Medical School, 225 Warren Street, Newark NJ 07102
Email: Yingda.xie@njms.rutgers.edu Tel: 973-972-2246

Clinical Project Manager:
Christie Eichberg
Medical University of South Carolina, 135 Rutledge Ave, Charleston, SC, USA 29425
Email: eichberg@musc.edu Tel: 843-792-5491

Data Management Head:
David Hom
Rutgers New Jersey Medical School, 185 South Orange Ave., Newark, New Jersey USA 07103
Email: homdl@njms.rutgers.edu Tel: 973-972-3221

***List of Institutions**:*

Medical University of South Carolina, Charleston, SC, USA (Coordinating Center)

Boston Medical Center, Boston, Massachusetts, USA (organizing site)

Johns Hopkins University, Baltimore, Maryland, USA (organizing site)

Foundation for Innovative New Diagnostics, Geneva, Switzerland (organizing site)

Rutgers New Jersey Medical School, Newark, New Jersey, USA (Data Coordinating Center and organizing site)

Infectious Diseases Institute – Makerere University, Kampala, Uganda (implementing site)

Kenya Medical Research Institute, Kisumu, Kenya (implementing site)

University of Cape Town, Cape Town, South Africa (implementing site)

Organization and Roles/Responsibilities

This is a multi-center study conducted by the TB Clinical Diagnostics Research Consortium. This study will include conduct of a research protocol carried out at more than one institution.

Medical University of South Carolina
The Medical University South Carolina (MUSC) will serve as the overall Coordinating Center. The Coordinating Center will be responsible for general oversight of conduct of the project, as well as monitoring and communication among all sites. No subjects will be enrolled at MUSC; MUSC personnel will not interact with study subjects. The Coordinating Center will ensure that each participating site has an active FWA with the OHRP, as well as appropriate regulatory approvals. The Coordinating Center will conduct study-specific training at implementing sites. The Coordinating Center will distribute study documents and ensure that the most current version of the protocol and any amendments are submitted at study sites for regulatory review/approval and implemented. The Coordinating Center will maintain regular communication with all study partners. In accordance with a written quality management plan, the Coordinating Center will conduct periodic in-person visits to implementing sites for the purpose of quality monitoring, review of regulatory documents, and assurance of study conduct in accordance with the protocol and procedures manuals. The Coordinating Center will be responsible for communication with the sponsor.

Boston Medical Center
Dr. Acuna-Villaorduna will provide scientific advice to the study team. He will have access only to de-identified data. He will not interact with study subjects.

John Hopkins University (JHU)
Dr. Yukari Manabe will provide scientific advice to the study team. She will have access only to de-identified data. She will not interact with study subjects.

Foundation for Innovative New Diagnostics (FIND)
Dr. Claudia Denkinger will provide expert scientific and technical advice to the study team and input into study design. She will have access only to de-identified data. She will not interact with study subjects.

Rutgers New Jersey Medical School (NJMS)
The Data Coordinating Center will be housed at Rutgers New Jersey Medical School (see Protocol Section 13). Data Coordinating Center personnel will have access only to de-identified data. They will not interact with study subjects.

Additionally, Drs. David Alland, Jerry Ellner, and Linda Xie will be involved in this study by providing expert scientific and technical advice to the study team, input into study design, and interpretation of investigational Xpert MTB/RIF Ultra test results. They will assist with data analysis, preparation of written study reports, and dissemination of study results to the scientific community. They will have access only to de-identified data. They will not interact with study subjects.

Implementing Sites
Under the direction of the local implementing site PI, and in compliance with the protocol and in accordance with Good Clinical Practice (GCP), the implementing sites will recruit, consent, enroll, and assess study participants and their specimens, as well as provide study data to the Data Coordinating Center. They may assist in development of the protocol and related documents as well as preparation of written study reports and dissemination of study findings.

2 BACKGROUND INFORMATION AND SCIENTIFIC RATIONALE

2.1 Background Information

Tuberculosis (TB) causes over 8 million cases and 1.3 million deaths per year. Over 95% of new TB cases and deaths occur in developing countries [1], and TB accounts for approximately 25% of all HIV/AIDS-related deaths worldwide, with almost 80% of deaths occurring in sub-Saharan Africa [20]. Likewise, a recent study showed that in resource-limited settings, TB accounts for approximately 40% of facility-based HIV/AIDS-related adult deaths, and that in almost half, TB was undiagnosed at the time of death[18]. Culture is the gold standard for TB detection, but remains restricted to higher levels of the health infrastructure because of expertise and equipment requirements. Furthermore, it takes weeks to obtain a result. Sputum smear microscopy is still the most widely used up-front diagnostic in most countries, but only detects about half of TB cases [2].

The Xpert® MTB/RIF assay on the GeneXpert platform (both from Cepheid, Sunnyvale, CA) has shown the potential of nucleic acid amplification tests to provide rapid, sensitive diagnosis and has now been rolled out in over 100 countries [3]. Xpert MTB/RIF can provide a result for TB detection and rifampin resistance on sputum within about two hours and requires minimal hands-on time [4]. Xpert MTB/RIF detects M. tuberculosis through a single copy target, namely the rpoB gene, using real-time PCR. The sensitivity for pulmonary TB detection has been demonstrated to be 98% for sputum smear-positive individuals and 67% for sputum smear-negative individuals, with a specificity of 99% over 27 studies with close to 10,000 participants. The performance characteristics for rifampin resistance detection are 95% sensitivity and 98% specificity [5, 6]. In 2010 the WHO endorsed the Xpert MTB/RIF assay for use as the initial diagnostic test in individuals suspected of multidrug-resistant TB or HIV-associated TB and in 2014 expanded the recommendation for use in all patients if resources allow [6]. Urine has also been tested using Xpert MTB/RIF, providing a useful diagnostic yield among patients with advanced immunodeficiency [21,22].

Despite these promising results, Xpert MTB/RIF also has demonstrated limitations. Its sensitivity in HIV-infected patients, while much improved over sputum smear microscopy, is estimated to be about 10% lower than for HIV-negative patients [5]. Further, Xpert MTB/RIF tests on urine can detect TB that is not detected by Xpert MTB/RIF in sputum of HIV infected individuals [19-21]. Similarly, Xpert MTB/RIF sensitivity is decreased in other paucibacillary disease (e.g. early presentation of pulmonary TB, in children) [7-9]. The suboptimal sensitivity in these important subgroups results in a substantial amount of empiric treatment including overtreatment that undermines the impact of the test [10,11].

Furthermore, issues have been demonstrated in the sensitivity of rifampicin resistance detection, particularly related to heteroresistance and silent mutation detection [12]. That being said, initial concerns around specificity of rifampicin resistance detection have been largely refuted, as poor clinical outcomes have been associated with Xpert rifampin resistant and phenotypically sensitive isolates. In this context the reference standard of phenotypic drug susceptibility testing has been put into question [13, 14]. Operational issues with Xpert MTB/RIF have also become manifest, resulting in increased error rates and module failure in settings where environmental temperature and/or humidity are high and cannot be controlled and where abundant dust is present [15].

Overall, the roll out of Xpert MTB/RIF has been successful but there is need for improvement both in performance and in operational characteristics. The recently developed Xpert MTB/RIF Ultra (Cepheid) addresses many of these issues. Xpert MTB/RIF Ultra, like its predecessor Xpert MTB/RIF, is an integrated cartridge-based nucleic acid amplification test that is compatible with the existing GeneXpert platform. To improve assay sensitivity for detection of *M. tuberculosis*, the Xpert MTB/RIF Ultra incorporates two different multicopy amplification targets and a larger DNA reaction chamber than the standard Xpert MTB/RIF. Xpert MTB/RIF Ultra also incorporates fully nested nucleic acid amplification, more rapid thermal cycling, and improved fluidics. Xpert MTB/RIF Ultra has a limit of detection of 10 bacterial colony forming units in early feasibility studies (compared to 131 colony forming units for Xpert MTB/RIF) [16]. In blinded testing of 32 previously frozen sputum samples, sensitivity for detection of *M. tuberculosis* in smear-negative/culture-positive samples was 94% (15/16) for Xpert MTB/RIF Ultra vs. 63% (10/16) for Xpert MTB/RIF. Sensitivity of Xpert MTB/RIF Ultra was 100% (6/6) for smear-positive/culture-positive samples, and specificity was 100% (10/10) for samples that were culture-negative for *M. tuberculosis*. To improve accuracy of rifampin resistance detection, the Xpert MTB/RIF Ultra incorporates melting temperature-based analysis instead of real-time PCR. Specifically, four probes identify rifampin resistance mutations in *rpoB* by shifting the melting temperature away from the wild type reference value. Analytic studies have demonstrated that these probes reliably distinguish between wild type (rifampin susceptible) and mutant (rifampin resistant) *rpoB* sequences, and that the melting temperature profiles are robust over a wide range (10^8 to 5 colony forming units/ml) of *M. tuberculosis* concentrations. Compared with Xpert MTB/RIF, Xpert MTB/RIF Ultra demonstrates improved detection of rifampin susceptible and resistant mixtures of bacteria.

The initial phase of this study tested the hypotheses that a single Xpert MTB/RIF Ultra test has sensitivity for TB case detection that is non-inferior to the sensitivity of a single Xpert MTB/RIF test. Results from the initial phase showed that, for tuberculosis case detection, the sensitivities of the Ultra test and the Xpert MTB/RIF test were 62.8% and 46.0%, respectively (difference of +17%, 95%CI +10, +24) among smear-negative culture-positive participants; 88.3% and 82.9%, respectively (difference of +5.4%, 95%CI +3.3, +8.0) among all culture-positive participants; and 89.6% and 76.5%, respectively (difference of +13%, 95%CI +6.4, +21) among HIV-infected participants [23]. Thus the Ultra test sensitivity for TB case detection was non-inferior and superior to that of conventional Xpert MTB/RIF overall, among smear-negative TB patients, and among HIV-infected TB patients. However, specificity of the Ultra test for TB case detection was only 95.6%, whereas specificity of Xpert MTB/RIF was 98.3% (difference -2.7%, 95%CI -3.9, -1.7).

Apparent “false-positive” Ultra results (i.e. a positive Ultra result without any culture positive for *M. tuberculosis*) were associated with a history of prior treatment for tuberculosis. Among participants with versus without prior tuberculosis treatment, Ultra specificities were 93.5% versus 96.4%. Furthermore, among participants with a history of prior tuberculosis treatment, the Ultra specificity reduction was greatest for those who had recently completed their TB treatment. With regard to rifampin drug susceptibility testing, Ultra provided interpretable rifampin drug susceptibility testing results for 86.0% of evaluable participants, whereas Xpert MTB/RIF provided results for 84.8%. For detection of rifampin resistance, Ultra sensitivity was 94.9% and Xpert MTB/RIF sensitivity was 95.4%, for a difference

favoring Xpert of -0.6% (95% CI -3.2, +1.6). Ultra specificity was 98.4% and Xpert MTB/RIF specificity was 98.1%, for a difference favoring Ultra of +0.3% (95% CI -0.7, +1.5).

Since the completion of the initial phase, the Ultra assay (version 2) has been optimized to extend the kit stability and to better align the range set for the semi-quantitative results with Xpert MTB/RIF G4 assay based on customer requests. The expansion phase will establish if there is any difference in current assay functionality in comparison to the lots utilized in the initial phase due to these changes.

The initial phase of the study did not explore the natural history or biological basis of apparent "false-positive" Ultra results. Interestingly, however, a recent study demonstrated the presence of *M. tuberculosis* mRNA in respiratory specimens as well as positron emission tomography/CT imaging response patterns consistent with active TB in a substantial proportion of adult patients with pulmonary tuberculosis after a standard 6-month treatment plus one year of follow-up, including patients with durable cure and other patients who later developed recurrent TB [24]. Several research teams recently identified blood RNA signatures that correlate with the risk of progression to active TB and with response to TB treatment [25-27].

We speculate that in our initial phase study of Ultra, most instances of Ultra-positive/no culture positive reflected the presence in sputum of non-cultivatable intact *M. tuberculosis* bacilli originating from the participant's lower respiratory tract, and that such individuals are at increased risk for development of overt TB disease. A better understanding of the natural history of apparent "false-positive" Ultra tests is critical for clinical decision-making based on test results. More broadly, understanding the biology and clinical implications of nucleic acid amplification test-positive/no culture positive results is important to the TB field since other TB diagnostic tests in development use the same multi-target approach as Ultra for optimizing sensitivity for *M. tuberculosis* detection. A better understanding of the biology of this scenario may shift our current concepts of disease cure and aid in development of better biomarkers for TB disease activity.

2.2 Rationale

Early analytical data for the Xpert MTB/RIF Ultra test show promising improvements in the lower limit of *M. tuberculosis* detection and in the sensitivity of rifampin resistance detection in sputum, compared with the conventional Xpert MTB/RIF test. Preliminary results from the initial phase of the clinical study support that the sensitivity of Xpert MTB/RIF Ultra for TB case detection is non-inferior and superior to that of the conventional Xpert MTB/RIF test, overall, among smear-negative TB pulmonary TB patients, and among HIV/TB patients. However, specificity was somewhat lower than expected, especially among patients with a history of prior TB treatment. The expansion phase of this study addresses the next critical questions along the Ultra diagnostic assay evaluation pathway. The expansion phase will assess whether, for TB case detection, Ultra sensitivity is not meaningfully worse than that of the accepted gold standard of liquid culture (non-inferiority). The expansion phase will also evaluate the diagnostic accuracy of the revised versus original Ultra assay version. To address these questions the expansion phase will accrue additional participants into the

Case Detection Group and will incorporate the same study procedures as used in the initial study phase. This strategy will allow data from the two phases to be aggregated, and will be efficient from an operations perspective. Importantly, the expansion phase will also characterize the natural history of participants with apparent “false-positive” Ultra results and will explore the biology of that scenario by incorporating, for a subset of study participants, longitudinal follow-up for 12 months as well as periodic sampling of sputum for microbiology studies and blood for host gene expression studies.

2.3 Potential Risks and Benefits

This is a diagnostic accuracy study. This study does not include TB treatment, and study staff will not be involved in clinical decision-making. This study includes performance of in vitro TB diagnostic tests applied to sputum. One test – the Xpert MTB/RIF Ultra (revised version 2) – is investigational, and the other sputum tests are conventional and considered standard of care for TB diagnosis. This study also includes HIV testing, which is WHO standard of care for TB suspects [1]. During the study, all tests will be performed by trained technologists according to strict procedures with careful quality management. Results of conventional tests (i.e. sputum smear microscopy, mycobacterial culture and drug susceptibility testing, and Xpert MTB/RIF) may be reported by designated study staff to a participant’s routine (non-study) clinical care provider. The Xpert MTB/RIF Ultra test (revised version 2 under study here) has been endorsed by the WHO for initial testing of all TB suspects [30,31], and has received the Conformité Européene (CE) marking. For each implementing site, that site’s IRB/ethics committee will provide guidance as to whether Ultra test results may or may not be provided to that site’s participants’ routine (non-study) clinical care providers for potential use in clinical care decision-making.

2.3.1 Potential Risks

There are minimal risks associated with this study.

The main medical risk is that associated with collection of blood specimens. There are minimal risks associated with peripheral vein phlebotomy to obtain blood; these include mild, temporary discomfort at the needle insertion site, and occasionally bleeding and/or bruising. Very rarely, patients may experience vasovagal syndrome during phlebotomy. Vasovagal reactions may include diaphoresis, nausea, syncope, and rarely fainting. There are minimal risks associated with obtaining spontaneously expectorated sputum (for mycobacteriology studies).

The other main risk is that of confidentiality. Confidentiality will be protected through the measures described in Section 12.

2.3.2 Known Potential Benefits

Knowledge gained from this study may benefit society by improving TB diagnosis in the future. Study participants may directly benefit from the study because they will be provided with a more comprehensive TB diagnostic assessment than may be routinely available to them.

Given the minimal risks associated with this study and the potential benefits to society and individuals, the benefits outweigh the risks. As for any clinical study, there is a possibility of unknown and unforeseen risk; that possibility is small for this study. If unforeseen risks are recognized during the study, then the sponsor, IRBs/ethics committees, and participants will be provided with relevant information.

3 OBJECTIVES

Primary:

- To estimate and compare the sensitivity of a single Xpert MTB/RIF Ultra test vs. that of a single MGIT liquid culture for detection of culture-positive pulmonary TB in new TB suspects
- To compare Xpert MTB/RIF Ultra accuracy estimates using revised vs. original assay definition file cut-offs

Secondary:

- To estimate and compare the specificity of two Xpert MTB/RIF Ultra tests vs. that of a single MGIT liquid culture for detection of culture-positive pulmonary TB in new TB suspects
- To estimate and compare the sensitivity and specificity of the Xpert MTB/RIF Ultra test vs. standard Xpert MTB/RIF for detection of culture-positive pulmonary TB in new TB suspects overall, by sputum smear microscopy status, and by HIV status
- To estimate and compare the sensitivity and specificity of the Xpert MTB/RIF Ultra test vs. standard Xpert MTB/RIF for detection of rifampin resistance
- To determine Xpert MTB/RIF Ultra test operating characteristics including proportion of tests with a non-determinate result
- To describe current routine clinical care practices regarding initiation of TB treatment
- To model the potential health impact and cost-effectiveness of TB diagnosis using the Xpert MTB/RIF Ultra
- To estimate the negative predictive value of Xpert MTB/RIF Ultra

Exploratory:

- To explore the relationship between Xpert MTB/RIF Ultra positivity and clinical, and microbiological markers of TB disease activity in participants whose enrollment cultures are negative for *M. tuberculosis*

4 STUDY DESIGN

This will be a multicenter diagnostic accuracy study in which the performance of a sputum rapid molecular diagnostic test (Xpert MTB/RIF Ultra) will be assessed using the reference gold standard of sputum culture.

The study is mainly cross-sectional with limited longitudinal follow-up (two months) for most participants; certain participants will have follow-up for 12 months (prospective cohort component).

For the initial study phase, two groups of participants were recruited -- the larger 'Case Detection Group' was comprised of individuals undergoing evaluation for TB who were not already known to have TB, and a smaller 'Drug Resistant TB Group' was comprised of individuals at high risk of drug-resistant TB. For the expansion study phase, only the Case Detection Group will be open for enrollment. After an informed consent process, sputum will be collected four times over about two days, and blood will be collected. All participants will have a follow-up visit approximately 7 days later for interview only, and a follow-up visit approximately two months later. Certain participants (Section 5.4), based on mycobacteriology test results at enrollment, will be followed in the study for 12 months in order to explore the relationship between Ultra positivity and clinical and microbiological markers of TB disease activity.

TB treatment is not a component of this study. Study staff will not be engaged in TB treatment or decisions about TB treatment for study participants. However, whether each participant is or is not started on TB treatment by his/her routine clinical care provider will be recorded for study purposes in order to inform the potential impact of the investigational test.

Information about HIV status of participants will be obtained in this study in order to provide estimates of test performance in the important sub-group of HIV-positive TB suspects, and also since HIV testing is considered standard of care for TB suspects.

4.1 Study Endpoints

4.1.1 Primary

- Sensitivity of a single Xpert MTB/RIF Ultra and of a single MGIT liquid culture in participants with culture-positive pulmonary TB
- Xpert MTB/RIF Ultra diagnostic accuracy using revised vs. original assay definition cut-offs. Diagnostic accuracy estimates will include sensitivity and specificity for case detection, and sensitivity and specificity for detection of rifampin resistance.

4.1.2 Secondary

- Sensitivity of two Xpert MTB/RIF Ultra tests and of a single MGIT culture in participants with culture-positive pulmonary TB

- For Xpert MTB/RIF Ultra and for Xpert MTB/RIF, sensitivity and specificity for detection of rifampin resistance

- Sensitivity and specificity of Xpert MTB/RIF Ultra and of Xpert MTB/RIF in participants with sputum culture-positive pulmonary TB. Results will be analyzed for the overall case detection group, stratified by sputum smear microscopy, and stratified by HIV status.

- For Xpert MTB/RIF Ultra and for Xpert MTB/RIF, proportion of test attempts that do not yield a determinate result

- For participants with culture-positive pulmonary TB, the incremental yields of a 2nd and a 3rd Xpert MTB/RIF Ultra test

- Proportion of participants on TB treatment at 7 days after enrollment, and proportion of participants on TB treatment at 2 months after enrollment

- Days from enrollment to initiation of TB treatment

- Vital status at 2 months after enrollment

- Mathematically modeled TB prevalence, TB incidence, TB mortality, and MDR-TB prevalence under an Xpert MTB/RIF Ultra scenario and also under an Xpert MTB/RIF scenario that is based on World Health Organization recommendations

- Calculated disability-adjusted life years saved and incremental cost effectiveness ratio under an Xpert MTB/RIF Ultra scenario and also under an Xpert MTB/RIF scenario that is based on World Health Organization recommendations.

- Negative predictive value (NPV) of Xpert MTB/RIF Ultra and of Xpert MTB/RIF, as observed in the study population, and as calculated under other TB epidemiology scenarios.

4.1.3 Exploratory

- Number and proportion of participants with baseline Ultra-positive/culture-negative results and of negative control participants who become culture positive for *M. tuberculosis* during follow-up

- Expression of *M. tuberculosis* mRNA transcripts in sputum at baseline

- Host (human) gene expression in whole blood at baseline, and change over time

4.2 Timeline (projected)

Months 1-3: IRB approvals, import permits

Month 4: Study-specific training

Months 5-20: Enrollment and enrollment testing

Months 7-44: Follow-up and follow-up testing

Months 44-48: Data analysis and dissemination

4.3 Blinding

All operators performing Xpert MTB/RIF Ultra tests will be blinded to the results of sputum smear microscopy, conventional Xpert MTB/RIF testing, mycobacterial cultures, and clinical status of participants. Operators performing and interpreting exploratory tests (*M. tuberculosis* RNA expression in sputum, host RNA expression in whole blood) will be blinded as to clinical and microbiological classification of participants.

5 SUBJECT SELECTION AND ENROLLMENT

5.1 Selection of the Study Population and Recruitment

Individuals who have symptoms consistent with pulmonary TB presenting to participating centers will be asked to participate (see inclusion/exclusion criteria).

Individuals will be recruited at outpatient clinic settings and inpatient hospital settings. Individuals will be asked by non-study clinicians or staff if they would be interested in participating in the study. Interested individuals will be referred to study personnel for additional information and screening, if appropriate. HIV-positive individuals and HIV-negative individuals will be included in this study.

5.2 Inclusion/Exclusion Criteria

To be eligible for study enrollment during the expansion phase, participants must meet all of the Inclusion Criteria and none of the Exclusion Criteria.

5.2.1 Inclusion Criteria

- Age 18 years or above;
- Provision of informed consent;
- Willingness to provide 4 sputum specimens at enrollment;
- Willingness to have study follow-up visits
- Clinical suspicion of pulmonary TB (including cough ≥2 weeks and at least 1 other symptom typical of TB);

5.2.2 Exclusion Criteria

- Receipt of *any* anti-TB treatment within 6 months prior to enrollment;
- Participants for whom, at the time of enrollment, the follow-up visit is judged to be poorly feasible (e.g. individuals planning to relocate).

5.3 Early Withdrawals

Participants who have provided consent and who are enrolled, but who at enrollment do not provide a total of 3 sputum specimens of sufficient volume will be classified as early withdrawals. Sufficient volume is defined as 2 ml or greater for each of sputa S1, S2, and S3.

5.4 Selection of Participants for Follow-up over 12 Months

Based on results of mycobacteriology testing performed at enrollment, certain participants will have study follow-up over twelve months (in-person visits at Months 2, 6, and 12). The following operational definitions will be used to classify participants based on enrollment test results:

Discordant participant: any enrollment Ultra test result of “MTB detected” AND no MGIT culture with Mtb growth at week 6 after inoculation AND no LJ culture positive for growth consistent with Mtb at week 6 after inoculation. Smear and Xpert MTB/RIF results are not components of the definition.

Eligible for Negative Control Group: no enrollment sputum smear microscopy positive for AFB AND Sputum 1 Xpert MTB/RIF result “MTB not detected” AND all (Sputum 1, Sputum 2, and Sputum 3) enrollment Ultra tests with result of “MTB not detected” AND two MGIT cultures evaluable (not contaminated) and negative for growth at week 6 after inoculation.

Eligible for Positive Control Group: any enrollment Ultra test result of “MTB detected” AND any MGIT culture positive for Mtb within 6 weeks after inoculation. Smear and Xpert MTB/RIF results are not components of the definition.

Controls will be selected such that approximately half are HIV-infected and approximately half are not HIV-infected, and controls will be approximately evenly distributed by site. Target total number of negative controls is 60 (estimated ratio of 2 negative controls per each discordant participant), and target total number of positive controls is 30 (estimated ratio 1 positive control per each discordant participant).

5.5 Voluntary Withdrawal by a Participant

Participants may withdraw voluntarily from study participation at any time. Given the nature of the study, there are no protocol-prescribed criteria for participant withdrawal. Participants who withdraw will be referred to the appropriate local health service for TB evaluation and care. If positive for *M. tuberculosis*, results of conventional microbiological tests (e.g., sputum smear microscopy, mycobacterial cultures, and Xpert MTB/RIF) will be reported to appropriate local health authorities in accordance with local public health reporting regulations. Study data for participants who withdraw consent will be destroyed and results will not be used for any final analyses.

5.6 Termination of the Study

Given the nature of the study, termination due to safety or other reasons is not anticipated. However, should the study be terminated, participants will be referred to the appropriate local health service for TB evaluation and care. If positive for *M. tuberculosis*, results of conventional microbiological tests (i.e., sputum smear

microscopy, mycobacterial cultures, and Xpert) will be reported to appropriate local health authorities in accordance with local public health reporting regulations.

6 STUDY PROCEDURES

All study procedures will be performed according to written Manuals of Procedures.

6.1 Screening Procedures

Written informed consent will be obtained before any study-specific procedures are performed.

The following will be performed to assess eligibility:

- Provision of informed consent
- Targeted assessment of TB signs/symptoms
- Targeted assessment of prior TB history and treatment
- Review of information about age
- Assessment of willingness and ability to comply with study procedures

6.2 Enrollment/Baseline (Day 1 and Day 2)

6.2.1 Clinical

The following evaluations will be performed:

- Collection of demographic information
- Targeted medical history
- HIV testing, unless any one or more of the following are present: written results of a positive HIV antibody test, written results of a positive HIV viral load, documentation in the medical record of positive HIV status by a treating clinician, immediate/verifiable documentation of HIV negativity within the preceding one month or within a timeframe specified by local health authorities for HIV re-testing. HIV testing can be performed using any test method approved by local health authorities. Depending on the test method used, this test will require approximately one milliliter of saliva (rapid oral test), or up to approximately 5 ml of blood.
- Blood for CD4 lymphocyte test for HIV-positive participants (approximately 5 ml), unless immediate/verifiable documentation of CD4 testing within the preceding 90 days is present.
- Blood drawn for host RNA expression studies. Up to approximately 10 ml of blood will be required, drawn into a Paxgene (or study-specified equivalent) tube.

- Blood drawn (up to approximately 10 ml) for investigational in vitro serum MTB antigen assays, only at sites where there is sufficient capacity to perform the work.

- Urine collected (up to approximately 30 ml) for investigational in vitro serum MTB antigen assays, only at sites where there is sufficient capacity to perform the work.

- Participants will be asked to provide four sputum samples (S1, S2, S3 and S4) over Days 1 and 2. Each specimen should be 2 ml or greater in volume. The intent is for all samples to be collected before the subject starts TB treatment. The intent is for Sputum S3 to be a morning specimen.

6.2.2 Laboratory testing of sputa S1, S2, S3, S4

Sputum 1 (S1)

- Direct fluorescence smear microscopy
- Direct Xpert MTB/RIF Ultra (version 2) test
- Direct Xpert MTB/RIF test

Sputum 2 (S2)

- NALC-NaOH decontamination followed by:
  - Xpert MTB/RIF Ultra (version 2) test
  - Fluorescence smear microscopy
  - Lowenstein Jensen culture
  - MGIT liquid culture (with extended incubation for discordant participants and negative controls)

Sputum 3 (S3)

- NALC-NaOH decontamination followed by
  - Xpert MTB/RIF Ultra (version 2) test
  - Fluorescence smear microscopy
  - Lowenstein Jensen culture
  - MGIT liquid culture (with extended incubation for discordant participants and negative controls)

Sputum 4 (S4)

This specimen will be used for *M. tuberculosis* gene expression studies, and may be used for additional tests to detect TB. This sputum specimen should be processed promptly with Trizol (or study-specified equivalent product) per the Manual of Procedures, and stored frozen.

For all participants having a culture that is positive for *M. tuberculosis*, one *M. tuberculosis* isolate will undergo phenotypic testing for rifampin and isoniazid resistance using MGIT liquid culture. *M. tuberculosis* DNA will be purified and the rifampin resistance domain of the *M. tuberculosis rpoB* gene will be DNA sequenced for a) participants with rifampin resistance by any study method (Ultra, Xpert MTB/RIF, or phenotypic); and b) participants in whom there is no determinate Ultra

rifampin result. *M. tuberculosis* isolates may also be characterized by other methods including whole genome sequencing.

For participants that have an Xpert MTB/RIF Ultra result that indicates MTB is detected and whose cultures are positive only for growth of mycobacteria that is not *M. tuberculosis*, identification of the cultured mycobacteria will occur to the species level. In addition, to assess for the presence of a mixed infection (i.e. non-tuberculous mycobacterium plus *M. tuberculosis*) these cultures will be tested using the Xpert MTB/RIF Ultra test version 2.0 and the Xpert MTB/RIF test, and may also be tested using additional molecular methods as detailed in the Manual of Procedures.

All Xpert MTB/RIF Ultra cartridges for which the test result was 'MTB detected' will be frozen and temporarily retained for possible batch shipment to the Alland Laboratory at Rutgers New Jersey Medical School in the United States for detailed molecular analyses of bacterial nucleic acid to understand any discordant cartridge results.

6.3 Follow-up Visits

6.3.1 Day 7 Follow-Up Visit

All participants will have a follow-up visit at approximately day 7 after enrollment (visit window 7 to 14 days after enrollment). A targeted interval medical history focused on TB treatment will be performed. This visit may be conducted in-person or telephonically.

6.3.2 Month 2 Follow-Up Visit

All participants will have a follow-up visit at approximately two months after enrollment (visit window 42 days after enrollment to up to 70 days after enrollment). This visit will be in-person for discordant participants, negative controls, and positive controls. This visit may be conducted in-person or telephonically for all other participants.

6.3.2.1 Month 2 visit for discordant participants and negative controls

This visit will be in-person. The following will be performed:

- Targeted interval medical history focused on vital status and TB treatment
- A urine specimen will be collected (approximately 40 ml) followed by:
    - Xpert MTB/RIF
    - Urine lipoarabinomannan lateral flow assay (LAM)
    - Potential additional in vitro diagnostic tests for TB
- One spot sputum for NALC-NaOH decontamination followed by
    - Fluorescence smear microscopy
    - MGIT liquid culture

  - MGIT liquid culture with growth supplement for participants who have not started TB treatment (note: as this test is exploratory and requires reagents that may not be routinely available, failure to perform this test will not be considered a protocol deviation)
  - Xpert MTB/RIF Ultra version 2
  - Xpert MTB/RIF
- Blood will be collected for host gene expression studies (up to approximately 10 ml into Paxgene tube or study-specified equivalent product)

6.3.2.2 Month 2 visit for positive controls

This visit will be in-person. The following will be performed:

- Targeted interval medical history focused on vital status and TB treatment
- Blood (up to approximately 10 ml into Paxgene tube or study-specified equivalent product) will be collected for host gene expression studies
- One spot sputum for NALC-NaOH decontamination followed by:
  - Fluorescence smear microscopy
  - MGIT liquid culture
  - Xpert MTB/RIF Ultra version 2
  - Xpert MTB/RIF

6.3.2.3 Month 2 visit for all other participants

This visit may be in-person or telephonic. The following will be performed:

- Targeted interval medical history focused on vital status and TB treatment

6.3.3 Month 6 Follow-Up Visit (in-person)

6.3.3.1 Discordant participants and negative controls

The following will be performed:

- Targeted interval medical history focused on vital status and TB treatment
- One spot sputum for NALC-NaOH decontamination followed by:
  - Fluorescence smear microscopy
  - MGIT liquid
  - Xpert MTB/RIF Ultra version 2
  - Xpert MTB/RIF
- Blood will be collected for host gene expression studies (up to approximately 10 ml into Paxgene tube or study-specified equivalent product)

6.3.3.2 Positive controls

The following will be performed:

- Targeted interval medical history focused on vital status and TB treatment
- Blood will be collected for host gene expression studies (up to approximately 10 ml into Paxgene tube or study-specified equivalent)
- One spot sputum for NALC-NaOH decontamination followed by:
  - Fluorescence smear microscopy
  - MGIT liquid culture
  - Xpert MTB/RIF Ultra version 2
  - Xpert MTB/RIF

6.3.4 Month 12 Follow-Up Visit (in-person)

6.3.4.1 Discordant participants and negative controls

The following will be performed:

- Targeted interval medical history focused on vital status and TB treatment
- One spot sputum for NALC-NaOH decontamination followed by:
  - Fluorescence smear microscopy
  - MGIT liquid culture
  - Xpert MTB/RIF Ultra version 2
  - Xpert MTB/RIF
- Blood will be collected for host gene expression studies (up to approximately 10 ml into Paxgene tube or study-specified equivalent product)
- HIV test (if not known to be positive)

6.3.4.2 Positive controls

The following will be performed:

- Targeted interval medical history focused on vital status and TB treatment
- Blood (up to approximately 10 ml into Paxgene tube or equivalent study-specified product) will be collected for host gene expression studies
- HIV test (if not known to be positive)
- One spot sputum for NALC-NaOH decontamination followed by:
  - Fluorescence smear microscopy
  - MGIT liquid culture
  - Xpert MTB/RIF Ultra version 2
  - Xpert MTB/RIF

6.4 Specimen Handling, Storage, and Shipping

Handling of all clinical specimens will be described in a detailed Manual of Procedures. All laboratory studies will be performed at the implementing site except for those studies described below. Shipping, when required, will be performed in accordance with local and international regulations. Shipped specimens will be labeled only with a code, and not with any personal identifying information.

6.4.1 Xpert MTB/RIF Ultra cartridges for which the test result was 'MTB detected'

All Xpert MTB/RIF Ultra cartridges for which the test result was 'MTB detected' will be frozen and may be batch shipped to the Alland Laboratory at Rutgers New Jersey Medical School in the United States for detailed molecular analyses of bacterial nucleic acid to understand any discordant cartridge results. If, based on study results, it is determined that additional tests on these cartridges are NOT necessary, then these cartridges will not be shipped to the U.S. and they will be disposed of per the Laboratory Manual of Procedures.

6.4.2 Sputum for *M. tuberculosis* gene expression studies

Sputum samples will be assayed to detect *M. tuberculosis* mRNA targets including, but not limited to, *85B and hspX (acr)* [24]. If determined to be feasible, work will be performed at one or more of the implementing sites in Africa; alternatively the work will be performed in the Alland Laboratory at Rutgers New Jersey Medical School in the United States. Any information learned through these gene expressions studies is for research purposes only; results will not be provided to clinicians or participants and results will not be used for clinical care.

6.4.3 Blood for human gene expression studies

RNA sequencing and mRNA target amplification/detection strategies (such as quantitative real-time PCR) will be performed to identify and validate host RNA signatures for tuberculosis disease risk [24]. If determined to be feasible, work will be performed at one or more of the implementing sites in Africa; alternatively the work will be performed in the Alland Laboratory at Rutgers New Jersey Medical School in the United States. RNA will be extracted from whole blood. Any information learned through these gene expressions studies is for research purposes only; results will not be provided to clinicians or participants and results will not be used for clinical care.

6.4.4 Urine and blood (serum) for investigational *M. tuberculosis* antigen studies

Urine and blood (serum) will be assayed to detect MTB antigen targets, including but not limited to new-gerneration high-affinity lipoarabinomannan tests (LAM). If determined to be feasible, work will be performed at one or more of the implementing sites in Africa; alternatively the work will be performed in the Alland Laboratory at Rutgers New Jersey Medical School in the United States. Any information learned through these in vitro studies is for research purposes only; results will not be provided to clinicians or participants and results will not be used for clinical care.

6.4.5 Incubation of MGIT cultures with growth supplement

For discordant participants and negative controls who have not been started on TB treatment, a portion of the processed sediment from the month 2 sputum will be inoculated into a MGIT culture that will be supplemented with a culture supernatant-derived growth supplement in an effort to cultivate (in vitro) fastidious *M. tuberculosis* organisms. Any results or information gained from incubation of MGIT cultures with growth supplement is for research purposes only; results will not be provided to clinicians or participants and results will not be used for clinical care and normal reporting times will not be affected for clinical use.

6.5 Participant Compensation

Study subjects will be compensated in accordance with local norms for their time required for study participation and for transportation to and from study visits.

6.6 Collection of Cost Data

Cost data for performing conventional and investigational TB diagnostic tests will be collected through inspection of laboratory budgets and through direct observation and time analysis of staffing needs, work flow, and resources consumed.

7 INVESTIGATIONAL PRODUCTS

7.1 Investigational Products Description

The investigational product is the Xpert MTB/RIF Ultra cartridge version 2 (Cepheid). This cartridge is intended for the detection of *M. tuberculosis* in sputum as well as the detection of *M. tuberculosis* mutations associated with resistance to rifampin. The investigational product will be strictly accounted for, including receipt and inventory, storage, use during the study, and return or disposal, as detailed in the Manual of Procedures.

7.2 Acquisition

Procurement of the investigational product will be done through the manufacturer (Cepheid). It is the responsibility of each study site to maintain an updated inventory of the study materials and to inform the MUSC project coordinator immediately if additional materials need to be shipped.

7.3 Storage

Procedures for product storage and disposal will be described in a Manual of Procedures. Briefly, investigational products will be checked for quality at the time of receipt at the site. Investigational products will be stored according to the manufacturer's instructions; expired or unused investigational products will either be discarded or returned to the manufacturer, per company instructions.

7.4 Test Handling and Performance

Testing using the investigational products will be performed according to the manufacturer's instructions within the Manual of Procedures.

7.5 Export and Import Permits

It is expected that most countries will require import permits for receiving the investigational materials. Local sites are responsible for making import permit applications in a timely manner.

7.6 Quality Control Check for Incoming Shipments

Upon arrival of each new shipment of Xpert MTB/RIF Ultra cartridges, the sites will conduct and document an incoming quality check following the Manual of Procedures. New lots may only be used after this quality check is successfully passed.

8 SAFETY ASSESSMENT AND REPORTING

This study has minimal medical risks.

The main medical risk is that associated with collection of blood specimens. There are minimal risks associated with peripheral vein phlebotomy to obtain blood; these include mild, temporary discomfort at the needle insertion site, and occasionally bleeding and/or bruising. Very rarely, patients may experience vasovagal syndrome during phlebotomy. Vasovagal reactions may include diaphoresis, nausea, syncope, and rarely fainting. There are minimal risks associated with obtaining spontaneously expectorated sputum (for mycobacteriology studies) and saliva (for HIV testing in sites where oral testing is performed).

Any clinical adverse event that meets reporting requirements of the local IRB/IEC will also be reported to the MUSC Project Manager.

9 CLINICAL MONITORING

9.1 Site Monitoring Plan

The study investigators are responsible for ensuring that, for this study:

- human subjects' rights and well-being are protected;
- data are accurate, complete, and verifiable from source documents;
- the study complies with the protocol/amendment(s), sponsor requirements, ICH Good Clinical Practice guidelines, and applicable regulatory requirements.

The DMID-accepted Clinical Quality Management Plan activities for the investigative site will be implemented to verify the data quality, applicable regulatory documentation, and subject safety.

If the Sponsor deems necessary, monitoring visits by a sponsor-designated professional or monitor may occur at scheduled intervals prior to, during, and at study completion. Monitoring visits may include, but are not limited to, review of regulatory files, CRFs, informed consent forms, medical and laboratory reports, accountability of the investigational product, and protocol compliance. Study monitors will meet with investigators to discuss any problems and actions to be taken and document visit findings and discussions. The investigational site will provide direct access to all study-related sites, source data/documents, and reports for the purpose of internal monitoring and in the event of auditing by the sponsor and inspection by regulatory authorities.

In addition, MUSC staff may conduct auditing visits prior to, during, and at study completion.

10 STATISTICAL CONSIDERATIONS

10.1 Study Hypothesis

The initial study phase tested the hypotheses that for detection of *M. tuberculosis* in sputum, a single Xpert MTB/RIF Ultra test had sensitivity that is non-inferior to that of the Xpert MTB/RIF assay among *sputum smear-negative*, culture positive pulmonary TB patients.

The expansion study phase will test the hypothesis that, for detection of *M. tuberculosis* in sputum, a single Xpert MTB/RIF Ultra test will have sensitivity that is non-inferior to that of a single MGIT liquid culture among all culture positive pulmonary TB patients.

If a single Ultra test is found to have sensitivity (for case detection) that is not non-inferior to that of a single MGIT liquid culture, then we will test the hypothesis that two Ultra tests (performed on two sputa) have sensitivity for case detection that is non-inferior to that of a single MGIT liquid culture.

10.2 Analysis Plans

Data will be aggregated for all clinical study sites participating in DMID 15-0029. Aggregated as well as site-specific data will be analyzed. Study results will be reported in accordance with the STARD (Standards for Reporting of Diagnostic Accuracy) statement [17].

10.2.1 Primary Endpoint and Hypothesis

The hypothesis concerns TB case detection, and therefore will include only participants who provided at least 3 sputum specimens at enrollment. Non-inferiority of the sensitivity of Ultra to that of a single MGIT culture will be considered to be demonstrated if the lower limit of the 95% confidence interval for the difference between Xpert MTB/RIF Ultra sensitivity and MGIT culture sensitivity does not exceed 5.0%. Ultra sensitivity will be calculated based on the Sputum 1 Ultra result, and MGIT sensitivity will be calculated based on the Sputum 2 MGIT culture result, using the Sputum 3 culture results as the reference standard.

For expansion phase participants, Ultra diagnostic accuracy (sensitivity and specificity for TB case detection, and sensitivity and specificity for detection of rifampin resistance) will be calculated and compared using revised assay definition cut-offs and using original assay definition cut-offs.

The specificity of S1 Xpert MTB/RIF Ultra will be compared to that of S1 Xpert MTB/RIF in participants without tuberculosis (based on cultures). Analyses will be stratified by HIV status and by history of prior tuberculosis treatment.

10.2.2 Secondary Endpoints

If Ultra sensitivity based on Sputum 1 is inferior or not non-inferior/non-superior to that of Sputum 2 MGIT, then the analysis will be repeated using Ultra sensitivity calculated based on Sputum 1 Ultra plus Sputum 2 Ultra results (either or both positive is considered positive).

Secondary endpoints of Xpert MTB/RIF Ultra sensitivity and specificity for *M. tuberculosis* detection will be calculated using data from participants who provided 3 sputum specimens, using a per-participant analysis. Sensitivity and specificity of Xpert MTB/RIF Ultra and of Xpert MTB/RIF will be calculated and compared for the overall group, stratified by sputum smear microscopy status, and also stratified by HIV status.

Calculations of Xpert MTB/RIF Ultra sensitivity and specificity for detection of rifampin resistance will include all evaluable enrolled participants with culture-positive pulmonary TB that in addition have a determinate result (i.e. “susceptible” or “resistant”) for rifampin susceptibility testing by Xpert MTB/RIF Ultra; a per-participant analysis will be used.

Secondary analyses of Xpert MTB/RIF Ultra specificity will include (as TB positive) individuals a) who were culture-positive for *M. tuberculosis* on follow-up culture(s); b) individuals having exploratory test results consistent with active TB; c) individuals considered to be clinically diagnosed with TB by their treating clinician.

Proportion of participants started on TB treatment and days from study enrollment to initiation of TB treatment will be assessed based on information obtained at the follow-up visits. Results will be stratified by rapid test result combination. Of particular interest are comparisons of participants whose results are Xpert MTB/RIF negative/Xpert MTB/RIF Ultra positive versus participants whose results are Xpert MTB/RIF negative/Xpert MTB/RIF Ultra negative versus participants whose results are Xpert MTB/RIF positive/Xpert MTB/RIF Ultra positive; these comparisons will allow estimation of the potential impact of Xpert MTB/RIF Ultra on clinical decision making.

A non-determinate test result for *M. tuberculosis* detection will be considered as any testing attempt that does not yield a result of ‘MTB detected’ or ‘MTB not detected’; a non-determinate test result for rifampin resistance will be considered any testing attempt that does not yield a result of ‘RIF resistance detected’ or ‘RIF resistance not detected’. The number and proportion of non-determinate results will be calculated overall and stratified by classification of non-determinate result (e.g. invalid, error).

A dynamic compartmental model of TB will be used to assess the potential health and economic consequences associated with hypothetical roll-out of the Xpert MTB/RIF Ultra test. An Ultra scenario will be compared with an Xpert MTB/RIF scenario that is based on current WHO recommendations. Comparisons between these diagnostic strategies will be made using a calibrated mathematical model of TB that takes into account key features of TB natural history, transmission dynamics,

interactions with HIV infection, and treatment for HIV. Model simulations will be undertaken separately for each study setting, and over all study settings. Costs will be assessed using a health system perspective, expressed in US dollars, and calculated using an ingredients approach. Health outcomes of interest include TB prevalence, TB incidence, TB mortality, and MDR-TB prevalence. Health systems costs of interest include disability-adjusted life years, incremental cost-effectiveness ratio (ICER) computed over 10- and 20-year time horizons, and comparisons of ICERs to cost-effectiveness thresholds defined in reference to country-specific annual gross domestic product per capita. Sensitivity analyses will be conducted for model inputs with special focus on estimates of diagnostic algorithm sensitivity and specificity as well as empiric treatment.

10.3 Sample Size Considerations

For the expansion phase an additional 1200 participants will be enrolled, as calculated below.

The expansion study phase will test the hypothesis that, for detection of *M. tuberculosis* in sputum, Ultra sensitivity is non-inferior to of a single MGIT liquid culture among all culture positive pulmonary TB patients. The population of interest is the overall population of culture-confirmed pulmonary TB patients enrolled into the study.

We therefore carried out sample size calculations via Monte-Carlo Simulation, conservatively assuming a moderate correlation of 0.5 between the tests when testing samples from the same participant using two different testing methods. We generated 10,000 correlated binary data sets for each simulation, using a variety of input test sensitivity parameters. We selected the final sample size to show superiority in at least 80% of simulated data sets, with parameter estimates of Ultra sensitivity 88% and MGIT culture sensitivity 90% for case detection, with pre-specified margin of non-inferiority (delta) 5.0%. Once a sample size fulfilled this criterion, at least two additional simulations were run using the same parameter inputs to verify the stability of the simulation result. If results were unstable between repeated simulations, the process was repeated with an increased number of simulated data sets per simulation. The same was done if the simulation results did not calibrate well with input parameters or if the histograms of output parameters did not have a smooth distribution. For the above parameter estimates, 671 culture positive participants are required (Case Detection Group). Given that data are available from a total of 464 culture positive individuals who participated in the initial phase studies, therefore an additional 207 culture positive participants are calculated to be required. Further assuming that the prevalence of culture-positive TB in enrollees is 22%; and approximately 20% of enrolled participants will not submit three sputa and therefore will not be evaluable, a total of 1176 (rounded to 1200) additional enrolled participants is calculated to be required.

11 QUALITY CONTROL AND QUALITY ASSURANCE

Following a written DMID-accepted site quality management plan, the study investigators are responsible for conducting routine quality assurance and quality control activities to internally monitor study progress and protocol compliance. The principal investigator will ensure that all study personnel are appropriately trained and applicable documentations are maintained on site. The Data Coordinating Center will implement quality control procedures beginning with the data entry system and generate data quality control checks that will be run on the database. Any missing data or data anomalies will be communicated to the site for clarification and resolution.

The investigational site will provide direct access to all study-related records, source data/documents, and reports for the purpose of monitoring and auditing by the sponsor and inspection by local and regulatory authorities, if requested.

12 ETHICS/PROTECTION OF HUMAN SUBJECTS

12.1 Ethical Standard

This study will be conducted in compliance with the Declaration of Helsinki and Good Clinical Practice Guidelines in full conformity with the principles of the Belmont Report: Ethical Principles and Guidelines for the Protection of Human Subjects of Research of the National Commission for the Protection of Human Subjects of Biomedical and Behavioral Research (April 18, 1979) and codified in 45 CFR 46, 21 CFR 312, and/or ICH E6; 62 Federal Regulations 25691 (1997). Each participating institution will hold a current FWA issued by OHRP. All key study staff will be trained and certified in Good Clinical Practice.

12.2 Institutional Review Board

Each participating institution IRB or IEC will review and approve this protocol and associated informed consent documents. Any amendments to the protocol or consent materials will also be approved before they are implemented.

Informed consent will be obtained from individuals prior to performance of study-specific procedures. The informed consent process is described below.

12.3 Informed Consent Process

Informed consent of individual participants will be obtained prior to performance of study-specific procedures. The informed consent process will be initiated before a volunteer agrees to participate and will continue throughout the individual's study participation. The subject will sign or place his/her mark on the informed consent document for the main study before any procedures are undertaken for the study. A copy of signed informed consent documents will be given to the subject for their records. The consents will explain that subjects may withdraw consent at any time throughout the course of the study. Explanation and discussion of risks and possible benefits of this investigation will be provided to the subjects in understandable language. Adequate time will be provided to ensure that the subject has time to consider and discuss participation in the protocol. The consent forms will describe in detail the study procedures and risks/benefits associated with participation in the study. The rights and welfare of potential subjects will be protected by emphasizing that neither their access to medical care nor the quality of their care will be adversely affected if they decline to participate in this study.

12.4 Subject Confidentiality

Subject confidentiality is strictly held in trust by the participating investigators, their staff, and the sponsor and their agents. This confidentiality includes documentation, investigation data, subjects' clinical information, and all other information generated

during participation in the study. Results of conventional mycobacteriology tests (i.e., smear microscopy, cultures and Xpert MTB/RIF), will be reported to subjects' routine (non-study) clinical care provider, and, if positive for *M. tuberculosis*, will be reported to the local TB control program in accordance with local policy. Results of the Xpert MTB/RIF Ultra tests may be reported to subjects' routine (non-study) clinical care provider, if allowed by the local IRB/ethics committee. No other information concerning the study or the data generated from the study will be released to any unauthorized third party without prior written approval of the sponsor and the subject. The study monitor or other authorized representatives of the sponsor or governmental regulatory agencies may inspect all documents and records required to be maintained by the investigators, including but not limited to medical records (office, clinic, or hospital) of the subjects in this study. The clinical study site will permit access to such records.

Risks to confidentiality will be minimized. Each study subject will be assigned a unique identification number to be used on study forms, in the study database, and on study specimens. None of the study forms, study database, or study specimens will contain subjects' names or other information that could be used to identify them. The document linking subjects' unique identification numbers with participants' names and medical record numbers will be kept in a locked office, will not be accessible to personnel not associated with the study, and will be destroyed as soon as all data has been collected, cleaned, and the study database locked. Study forms will be maintained in a locked office and will not be available to personnel not associated with the study. All computers containing the study database will be password-protected and the study database will not be accessible by personnel not associated with the study.

13 DATA MANAGEMENT

13.1 Data Management Responsibilities

The Data Coordinating Center will be responsible for data collection and management. De-identified data will be shared with FIND during the study. A final de-identified dataset cleaned of errors will be provided to FIND at the end of the study.

13.2 Data Capture Methods

Teleform software will be used to create paper CRFs that will be completed onsite, scanned and uploaded using a secure method such as an ftp server, and read into the database via Teleform at the Data Coordinating Center. Teleform will digitally capture the CRFs, perform programmed quality control checks to validate the data, and index the forms. Forms will be manually reviewed for accuracy before the data is exported for storage in a Microsoft Access database. The data system includes password protection and internal quality checks to identify data that appear inconsistent, incomplete, or inaccurate.

13.3 Types of Data

Data for this study will include demographic information, clinical information, laboratory test results, and participant final diagnosis with respect to TB.

A detailed Data Management plan will describe the nature and timing of reports that will be used to track enrollments (overall and by site), early withdrawals, and other indicators of study progress and data quality.

13.4 Study Records Retention

Study documents will be retained for a minimum of 2 years after study completion, and longer if required by local regulations or sponsor. Study records will be retained until a letter approving destruction of the records is received from DMID; this letter will be kept with the regulatory file.

Each participating site will maintain appropriate records for this study, in compliance with ICH E6, Section 4.9, regulatory and institutional requirements for the protection of confidentiality of subjects. Each site participating in this study will permit authorized representatives of the sponsor(s), DMID, and regulatory agencies to examine (and when required by applicable law, copy) clinical records for the purposes of clinical site monitoring, quality assurance reviews, audits, and evaluation of study safety and progress.

13.5 Protocol Deviations

Protocol deviations occur when there is non-adherence to the Protocol and includes Informed Consent, enrollment, and other occurrences of non-adherence to the Protocol. Protocol deviations should be sent to the local IRB/IEC per the local IRB/IEC guidelines. Any protocol deviation that meets reporting requirements of the local IRB/IEC will also be reported with the same timeliness to the MUSC Clinical Project Manager. All deviations from the Protocol must be addressed in the study subject source documents. The documentation should include the reason(s) for the deviation and all attempts to prevent or correct the deviation. The site must complete a Protocol Deviation Form documenting each protocol deviation. A completed copy of the Protocol Deviation Form must be maintained in the regulatory file as well as in the subject's source documents.

14 LITERATURE REFERENCES

1. World Health Organization. Global Tuberculosis Control: WHO report 2013. Geneva: WHO, 2013.
2. Steingart KR, Ramsay A, Pai M. Optimizing sputum smear microscopy for the diagnosis of pulmonary tuberculosis. Expert Rev Anti Infect Ther 2007; 5(3): 327-31.
3. World Health Organization. WHO monitoring of Xpert MTB/RIF roll-out. . 2014.
4. Boehme CC, Nabeta P, Hillemann D, et al. Rapid molecular detection of tuberculosis and rifampin resistance. N Engl J Med 2010; 363(11): 1005-15.
5. Steingart KR, Schiller I, Horne DJ, Pai M, Boehme CC, Dendukuri N. Xpert(R) MTB/RIF assay for pulmonary tuberculosis and rifampicin resistance in adults. Cochrane Database Syst Rev 2014; 1: CD009593.
6. World health Organization. Policy update: Xpert MTB/RIF assay for the diagnosis of pulmonary and extrapulmonary TB in adults and children. 2014.
7. Sohn H, Aero AD, Menzies D, et al. Xpert MTB/RIF testing in a low tuberculosis incidence, high-resource setting: limitations in accuracy and clinical impact. Clin Infect Dis 2014; 58(7): 970-6.
8. Nicol MP, Workman L, Isaacs W, et al. Accuracy of the Xpert MTB/RIF test for the diagnosis of pulmonary tuberculosis in children admitted to hospital in Cape Town, South Africa: a descriptive study. Lancet Infect Dis 2011; 11(11): 819-24.
9. Peter JG, Theron G, Pooran A, Thomas J, Pascoe M, Dheda K. Comparison of two methods for acquisition of sputum samples for diagnosis of suspected tuberculosis in smear-negative or sputum-scarce people: a randomised controlled trial. The lancet Respiratory medicine 2013; 1(6): 471-8.
10. Theron G, Peter J, Dowdy D, Langley I, Squire SB, Dheda K. Do high rates of empirical treatment undermine the potential effect of new diagnostic tests for tuberculosis in high-burden settings? Lancet Infect Dis 2014.
11. Theron G, Zijenah L, Chanda D, et al. Feasibility, accuracy, and clinical effect of point-of-care Xpert MTB/RIF testing for tuberculosis in primary-care settings in Africa: a multicentre, randomised, controlled trial. Lancet 2014; 383(9915): 424-35.
12. Rufai SB, Kumar P, Singh A, Prajapati S, Balooni V, Singh S. Comparison of Xpert MTB/RIF with Line Probe Assay for Detection of Rifampin-Monoresistant Mycobacterium tuberculosis. J Clin Microbiol 2014; 52(6): 1846-52.
13. Van Deun A, Aung KJ, Bola V, et al. Rifampin drug resistance tests for tuberculosis: challenging the gold standard. J Clin Microbiol 2013; 51(8): 2633-40.
14. Somoskovi A, Deggim V, Ciardo D, Bloemberg GV. Diagnostic implications of inconsistent results obtained with the Xpert MTB/Rif assay in detection of Mycobacterium tuberculosis isolates with an rpoB mutation associated with low-level rifampin resistance. J Clin Microbiol 2013; 51(9): 3127-9.
15. Raizada N, Sachdeva KS, Sreenivas A, et al. Feasibility of decentralised deployment of Xpert MTB/RIF test at lower level of health system in India. PLoS One 2014; 9(2): e89301.
16. Helb D, Jones M, Story E, et al. Rapid detection of Mycobacterium tuberculosis and rifampin resistance by use of on-demand, near-patient technology. J Clin Microbiol 2010; 48(1): 229-37.
17. Bossuyt P, Reitsma J, Bruns D, et al. The STARD Statement for Reporting Studies of Diagnostic Accuracy: Explanation and Elaboration. Ann Intern Med 2003, 138, W1-12.
18. Gupta RK, Lucas SB, Fielding KL, et al. Prevalence of tuberculosis in post-mortem studies of HIV-infected adults and children in resource-limited settings: a systematic review and meta-analysis. AIDS 2015, 29:000–000.

19. Lawn SD, Kerkhoff AD, Burton R, et al. Rapid microbiological screening for tuberculosis in HIV-positive patients on the first day of acute hospital admission by systematic testing of urine samples using Xpert MTB/RIF: a prospective cohort in South Africa. BMC Medicine (2015) 13:192.
20. UNAIDS. Global report: UNAIDS report on the global AIDS pidemic 2013. 2013. http://www.unaids.org/sites/default/files/media_asset/UNAIDS_Global_Report_2013_en_1.pdf. 21.
21. Lawn SD, Kerkhoff AD, Vogt M,Wood R. High diagnostic yield of tuberculosis from screening urine samples from HIV-infected patients with advanced immunodeficiency using the XpertMTB/RIF assay. J Acquir Immune Defic Syndr 2012; 60:289–294.
22. Peter JG, Theron G, Muchinga TE, Govender U, Dheda K. The diagnostic accuracy of urine-based Xpert MTB/RIF in HIVinfected hospitalized patients who are smear-negative or sputum scarce. PLoS One 2012; 7:e39966.
23. Schumacher SG, Nabeta P, Boehme CC, Ellner J, Alland D, Dorman SE, Denkinger CM. A multicenter diagnostic accuracy study of the Xpert Ultra for tuberculosis diagnosis (Abstract 76LB). Conference on Retroviruses and Opportunistic Infections. Seattle, February 13-16, 2017.
24. Malherbe ST, Shenai S, Ronacher K, et al. Persisting positron emission tomography lesion activity and Mycobacterium tuberculosis mRNA after tuberculosis cure. Nat Med. 2016;22:1094-1100.
25. Zak DE, Penn-Nicholson A, Scriba TJ et al. A blood RNA signature for tuberculosis disease risk: a prospective cohort study. Lancet 2016;387:2312-22.
26. Berry MP, Graham CM, McNab FW, et al. An interferon-inducible neutrophil-driven blood transcriptional signature in human tuberculosis. Nature 2010;466:973-7.
27. Anderson ST, Kaforou M, Brent AJ, et al. Diagnosis of childhood tuberculosis and host RNA expression in Africa. N Engl J Med. 2014;370:1712-23.
28. American Thoracic Society, Centers for Disease Control and Prevention. Diagnostic standards and classification of tuberculosis in adults and children. Am J Respir Crit Care Med 2000;161:1376-95.
29. Theron G, Zijenah L, Chanda D et al. Feasibility, accuracy, and clinical effect of point-of-care Xpert MTB/RIF testing for tuberculosis in primary care settings in Africa: a multicentre, randomised, controlled trial. Lancet 2014;383:424-35.
30. WHO meeting report of a technical expert consultation: non-inferiority analysis of Xpert MTB/RIF Ultra compared to Xpert MTB/RIF. Geneva: World Health Organization; 2017 (WHO/HTM/TB/2017.04).
31. Compendium of WHO guidelines and associated standards: ensuring optimum delivery of the cascade of care for patients with tuberculosis. Geneva: World Health Organization; 2017. License: CC BY-NC-SA 3.0 IGO. (WHO/HTM/TB/2017.13)

Appendix A: Study Schedule

SCHEDULE OF EVENTS for each participant group

	Baseline				Day 7*				Month 2				Month 6				Month 12			
	Disc	Neg Ctrl	Pos Ctrl	Oth-er	Disc	Neg Ctrl	Pos Ctrl	Oth-er	Disc	Neg Ctrl	Pos Ctrl	Oth-er*	Disc	Neg Ctrl	Pos Ctrl	Oth-er	Disc	Neg Ctrl	Pos Ctrl	Oth-er
**Screening**	√															OFF STUDY; NO MONTH 6 VISIT				OFF STUDY; NO MONTH 12 VISIT
**Consent**	√																			
**Demographic info**	√																			
**Medical history**	√				√	√	√	√	√	√	√	√	√	√	√		√	√	√	
**Vital status**	Not applicable				√	√	√	√	√	√	√	√	√	√	√		√	√	√	
**Record review**	√																			
**HIV test**	√																√[1]	√[1]	√[1]	
**Blood for Paxgene/RNA**	√								√	√	√		√	√	√		√	√	√	
**Blood for CD4 count**	√ (if HIV-pos)								√[2]	√[2]	√[2]									
**Blood for serum assays**	√																			
**Urine**	√								√	√										

Appendix A: Study Schedule

Sputum Tests

	Baseline				Day 7*				Month 2				Month 6				Month 12			
	Disc	Neg Ctrl	Pos Ctrl	Oth-er	Disc	Neg Ctrl	Pos Ctrl	Oth-er	Disc	Neg Ctrl	Pos Ctrl	Oth-er*	Disc	Neg Ctrl	Pos Ctrl	Oth-er	Disc	Neg Ctrl	Pos Ctrl	Oth-er
**Direct smear**	Sp1															OFF STUDY; NO MONTH 6 VISIT				OFF STUDY; NO MONTH 12 VISIT
**Direct Xpert MTB/RIF**	Sp1																			
**Direct Ultra**	Sp1																			
**Conc smear**	Sp2, Sp3								√	√	√		√	√	√		√	√	√	
**Conc Xpert MTB/RIF**									√	√	√		√	√	√		√	√	√	
**Conc Ultra**	Sp2, Sp3								√	√	√		√	√	√		√	√	√	
**LJ culture**	Sp2, Sp3																			
**Routine MGIT culture**	Sp2, Sp3								√	√	√		√	√	√		√	√	√	
**MGIT extended incubation**	Sp2 Sp3	Sp2 Sp3																		
**MGIT culture + growth suppl[3]**									√[3]	√[3]										
**Sputum in Trizol**	Sp4																			

Disc : Discordant case * : may be performed by phone
1 (superscript) : perform HIV test at month 12 if not known to be HIV-positive
2 (superscript): perform CD4 testing for HIV-positive participants who did not have CD4 testing done at baseline, unless immediate/verifiable documentation of CD4 testing within the preceding 90 days is present
3 (superscript): perform only for participants who have not been started on TB treatment
